# Regulation of gene expression by the action of a fungal lncRNA on a transactivator

**DOI:** 10.1080/15476286.2019.1663618

**Published:** 2019-09-13

**Authors:** Petra Till, Christian Derntl, Daniel P Kiesenhofer, Robert L Mach, Debbie Yaver, Astrid R Mach-Aigner

**Affiliations:** a Christian Doppler laboratory for optimized expression of carbohydrate-active enzymes, Institute of Chemical, Environmental and Bioscience Engineering, TU Wien, Vienna, Austria; b Institute of Chemical, Environmental and Bioscience Engineering, TU Wien, Vienna, Austria; c Production Strain Technology, Novozymes Inc., Davis, CA, USA

**Keywords:** *Trichoderma reesei*, fungi, gene regulation, lncRNA, long non-coding RNA, *HAX1*, Xyr1

## Abstract

Long non-coding RNAs (lncRNAs) are crucial factors acting on regulatory processes in eukaryotes. Recently, for the first time in a filamentous fungus, the lncRNA *HAX1* was characterized in the ascomycete *Trichoderma reesei*. In industry, this fungus is widely applied for the high-yield production of cellulases. The lncRNA *HAX1* was reported to influence the expression of cellulase-encoding genes; interestingly, this effect is dependent on the presence of its most abundant length. Clearly, *HAX1* acts in association with a set of well-described transcription factors to regulate gene expression. In this study, we attempted to elucidate the regulatory strategy of *HAX1* and its interactions with the major transcriptional activator Xylanase regulator 1 (Xyr1). We demonstrated that *HAX1* interferes with the negative feedback regulatory loop of Xyr1 in a sophisticated manner and thus ultimately has a positive effect on gene expression.

## Introduction

In recent decades, long non-coding RNAs (lncRNAs) have emerged as crucial players in regulatory processes in various eukaryotic organisms. Similar to other non-coding RNAs (ncRNAs), lncRNAs do not code for proteins but function directly as RNAs. However, the group of lncRNAs is highly diverse and is distinguished from small functional ncRNA species by their size (more than 200 nt), rather than by certain common characteristics [1,2].

Recently, the first lncRNA was discovered and characterized in a filamentous fungus, namely, *Trichoderma reesei* [3]. This fungus is highly important for industrial purposes meeting human needs, as it produces large quantities of plant biomass degrading enzymes (PBDE) [4]. Most of the enzymes are cellulases, which are applied for manufacturing and processing of textile fabrics [5], food and feed [6], paper and pulp [7], and bio-ethanol [8,9]. In nature, *T. reesei* secretes those enzymes to degrade complex plant biopolymers, such as cellulose and xylan, to monomeric sugar molecules that can be easily metabolized. Thus, this fungus is capable of living on dead plant material [10]. However, this metabolic process is tightly regulated. When glucose is available as a carbon source, *T. reesei* stops the energetically demanding production of PBDE by so-called carbon catabolite repression (CCR) [11]. Moreover, PBDE expression strictly depends on transcriptional activation in the presence of inducer substances resulting from the hydrolysis of the complex plant biopolymers [12]. Both repression and activation of PBDE expression are regulated by transcription factors, ensuring the efficiency of this metabolic pathway [13–15]. The most important transactivator essential for the expression of almost all of the PBDE-encoding genes is the Xylanase regulator 1 (Xyr1) [16]. Xyr1 is a Gal4-like binuclear Zn-cluster protein that activates the expression of its target genes by binding to its DNA recognition sites at the promoter regions. According to earlier *in vitro* studies, the motif recognized by Xyr1 is GGCWWW [17], known as the Xyr1-binding site (XBS), as follows. *In vivo* studies indicate that GGC is the pivotal element to allow binding of Xyr1; hence, XBS bearing one mismatch in the WWW (XBS_mm_) may be functional sites. As in the case of its target genes, the level of expression of *xyr1* itself is controlled by CCR mediated by the Carbon catabolite repressor 1 (Cre1) [18]. In the promoter region of *xyr1* (−1033 to −1 bp), 8 Cre1-binding sites (SYGGRG [19]) are present [20]. The possibility of an auto-regulatory activity of Xyr1 was previously suggested [20]; however, to date, no evidence has been provided to support this possibility.

As recently reported, not only transcription factors but also the lncRNA *HAX1* are involved in the complex regulatory network of PBDE expression. It was found that versions with different lengths are present in different *T. reesei* strains [3]. Therefore, the *HAX1* versions were initially termed *HAX1*_QM6a_, *HAX1*_QM9414_ and *HAX1*_Rut-C30_ based on the strain in which they were predominant [3]. However, for more clarity, in this study, the versions are referred to as *HAX1*_262_, *HAX1*_299_ and *HAX1*_428_, indicating their lengths in nt. The discovery of lncRNAs of varying lengths in moderate PBDE-producing and -overproducing *T. reesei* strains suggested an association between *HAX1* length and the level of PBDE production. Indeed, this assumption was verified by overexpression of the different lengths of *HAX1* in either of the strains [3]. However, the regulatory mechanism governing this association has not been elucidated to date.

In general, the regulatory strategies of lncRNAs are highly diverse. These RNAs can act at proximal locations simply by blocking the accessibility of genes to the transcriptional machinery [1], or they might interfere with transcription by the formation of antisense transcripts [21]. In addition, lncRNAs can directly interact with DNA and RNA, as well as with proteins. Hence, these RNAs can serve as scaffolds or guides for different factors [22,23] and thus mediate such activities as coordinating DNA modification and packaging [24]. Some lncRNAs are also known to modulate the function and activity of their interaction partners. One example of this type of regulatory strategy is the vertebrate lncRNA Evf-2 [25]. In the case of *HAX1*; at this point, a trans-regulatory mode of action is evident. Furthermore, the sequence of all three *HAX1* versions comprises several XBS (i.e., 3 XBS and 2 XBS_mm_) located near the transcriptional start site of the shortest transcript version [3]. This finding suggests a direct interaction of *HAX1* and Xyr1. However, prior to this study, this possibility had not been investigated.

In this study, evidence for a physical interaction of the lncRNA *HAX1* and the key transactivator Xyr1 is provided. Moreover, a regulatory sequence in the *xyr1* promoter was identified as a currently undescribed DNA recognition site of Xyr1, which is involved in negative feedback regulation of the transactivator. The affinity of Xyr1 to the newly identified element in comparison to XBS was investigated, and the role of *HAX1* in the target gene preference of Xyr1 was determined. Based on the achieved results, a model for the molecular action of *HAX1* and its contribution to the regulation of gene expression in *T. reesei* is presented.

## Results

### *Fungal lncRNA* HAX1 *and the transactivator Xyr1 can bind*

**Figure 1.**
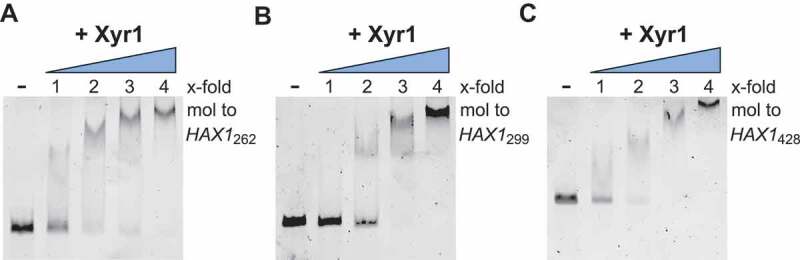
Analysis of the protein-RNA interaction of Xyr1 and *HAX1*. RNA-EMSAs using either version of the lncRNA *HAX1*, i.e., *HAX1*_262_ (A), *HAX1*_299_ (B) or *HAX1*_428_ (C), and increasing amounts of Xyr1. An up to 4-fold molar excess of Xyr1 relative to 1 µg of *in vitro* synthesized *HAX1* RNA was used.

As the recently discovered lncRNA *HAX1* has an unusually high number of XBS [3], a physical interaction of *HAX1* and the key transactivator Xyr1 can be hypothesized. To test this possibility, RNA electrophoretic mobility shift assays (RNA-EMSAs) were performed. To this end, the different versions of *HAX1* varying in length (i.e., *HAX1*_262_, *HAX1*_299_ and *HAX1*_428_ [3]) were synthesized *in vitro*, and Xyr1 was expressed heterologously. The addition of increasing amounts of Xyr1 to *HAX1* resulted in a shift of the RNA, depending on the Xyr1 concentration (Fig. 1). As a negative control, *HAX1* did not shift in the presence of bovine serum albumin (BSA) (Fig. S1). For all *HAX1* versions, a reduction in the signal of the unbound RNA was visible when equimolar amounts of Xyr1 were applied, and a 4-fold molar excess of Xyr1 resulted in a total shift (Fig. 1). However, slight differences could also be observed for the different versions of *HAX1*. When a 3-fold molar excess of the protein was used, the signal of the free RNA was still clearly visible in the case of *HAX1*_262_ (Fig. 1A), only slightly visible using *HAX1*_299_ (Fig. 1B) and, in the case of the longest version, fully abolished (Fig. 1C). In summary, it can be stated that the longer the version of *HAX1*, the lower amounts of Xyr1 are required to result in a complete shift. This finding indicates that Xyr1 can bind more efficiently to longer *HAX1* versions than to shorter ones. Based on this analysis, the physical interaction of *HAX1* and Xyr1 is clearly plausible, and length-specific effects were observed. Consequently, the regulatory interplay between *HAX1* and Xyr1 warrants further study.

### *Newly identified, repressive regulatory sequence in the* Xyr1 *promoter is also present in* HAX1*_428_*

**Figure 2.**
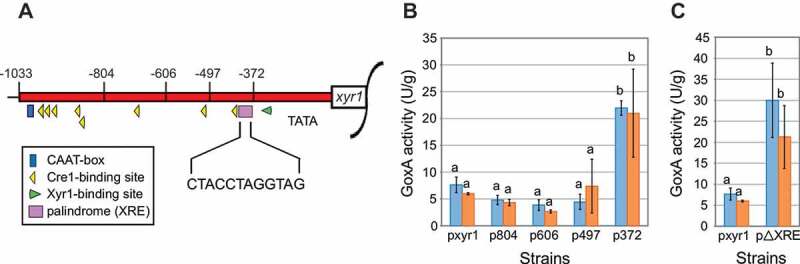
Analyses of altered versions of the *xyr1* promoter. (A) Schematic drawing of the *xyr1* promoter (red bar). Previously described regulatory elements, such as Cre1-binding sites (yellow triangles), a Xyr1-binding site (green triangle) and a CAAT-box (blue box), as well as the newly identified 12 bp palindromic DNA motif XRE (pink box), are marked. The approximate positions of truncations are indicated by the numbers given on top (bp from ATG). (B) GoxA activities of recombinant *T. reesei* strains expressing the reporter gene *goxA* under the control of one of the truncated promoter versions (p804, p606, p497, p372) or the full-length version (pxyr1). The strains were pre-grown and replaced to minimal medium without a carbon source (blue bars) or to minimal medium with sophorose (orange bars). Means of biological replicates are derived from independently generated strains (*n* = 2 for p804, p606, p497 and p372; *n* = 3 for pxyr1). Error bars depict standard deviations; ANOVA followed by a post-hoc Tukey multiple comparison test (*P* < 0.05) resulted in *F*(9;12) = 11.684. Different letters denote significant differences among compared data. A detailed itemization of the *P* values is appended in Table S3. (C) GoxA activities of recombinant *T. reesei* strains cultivated as described above and expressing the reporter gene *goxA* under the control of the full-length promoter (pxyr1) or the full-length promoter lacking XRE (pΔXRE). Means of biological replicates are derived from independently generated strains (*n* = 2 for pΔXRE; *n* = 3 for pxyr1). Error bars depict standard deviations; t-tests resulted in *t*(3) = 4.661, *p*< 0.01 for the cultivation without carbon source and *t*(3) = 3.326, *p*< 0.05 for the cultivation on sophorose. Different letters denote significant differences among compared data. A detailed itemization of the *P* values is appended in Table S3.

To identify sequence elements potentially involved in this regulatory process, the native *xyr1* promoter and four different truncations were fused to the commonly used reporter gene *goxA* from *Aspergillus niger* [26]. Compared to the 1033 bp full-length promoter, each of the four shortened versions (i.e., 804-, 606-, 497- and 372-bp long) lacks at least one known regulatory element (Fig. 2A). Most of the elements are Cre1-binding sites responsible for repression of *xyr1* under carbon catabolite repressing conditions. Only a single XBS (and no XBS_mm_) is present at this locus [20]. The promoter-reporter constructs were ectopically integrated into the fungal genome. The obtained strains (listed in Table S1) were incubated under two different conditions (i.e., availability of carbon source), and GoxA assays were performed. No significant differences in the GoxA activity compared to the strain carrying the full-length promoter could be observed for strains p804, p606, and p497 (Fig. 2B). Notably, a significant increase in GoxA activity was detected for p372 compared to all other strains (Fig. 2B). Hence, the presence of a DNA motif responsible for the negative regulation of *xyr1* expression in the sequence part −497 to −372 from the ATG was proposed. However, compared to p497, strain p372 only lacks a single Cre1-binding site. As it had been reported that Cre1 binds to double sites, it seemed unlikely that the presence of this site was the reason for the observed phenotype of strain p372. A sequence analysis revealed the presence of a 12-bp-long palindromic sequence (CTACCTAGGTAG), which is located at the end of this sequence. Given that palindromes and inverted repeats are often targets for binding regulatory factors [27,28], this motif was used for sequence alignment. Surprisingly, this palindrome is also present close to the 5ʹ end of the longest version of *HAX1* (i.e., *HAX1*_428_; see Fig. S2). To determine if the palindrome is a regulatory element, a version of the full-length *xyr1* promoter lacking only this motif was fused to the *goxA* gene, introduced into the fungal genome, and the obtained strains (pΔXRE, see Table S1) were studied as described above. As observed for strain p372 (compare to Fig. 2B), a significant increase in GoxA activity was detected for strain pΔXRE compared to the strain bearing the full-length promoter (pxyr1) (Fig. 2C). Therefore, we concluded that this motif conveys negative regulation of *xyr1* expression, and we termed it the Xyr1 regulatory element (XRE). Herewith we identified a previously unknown regulatory element responsible for the repression of *xyr1* expression, which is also present in *HAX1*_428_.

### Xyr1 can bind its own promoter via the XRE

**Figure 3.**
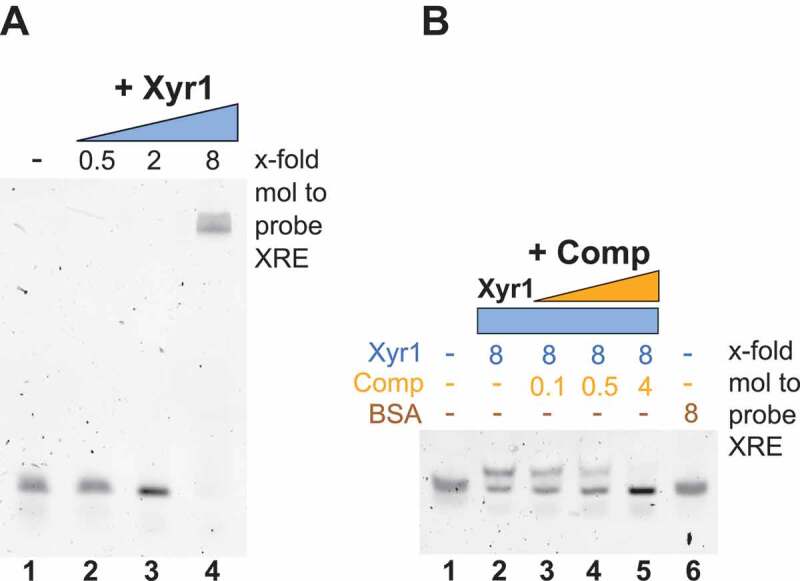
Analysis of the binding of Xyr1 to XRE. (A) EMSA using 33.4 ng of the FAM-labelled probe containing the XRE alone (lane 1) or together with a 0.5-fold, 2-fold or 8-fold molar excess of Xyr1 relative to the probe (lanes 2–4). (B) Control EMSA using 33.4 ng of the FAM-labelled probe alone (lane 1) or with an 8-fold molar excess of Xyr1 (lane 2) or with an 8-fold molar excess of Xyr1 and increasing amounts (0.1-fold, 0.5-fold and 4-fold molar excess) of the unlabelled probe (Comp) (lanes 3–5) or with an 8-fold molar excess of BSA (lane 6).

To investigate whether Xyr1 can bind to XRE, EMSA studies were performed. As a probe, a FAM-labelled 35 bp ds DNA fragment composed of the XRE and its adjacent genomic region was applied. Using an 8-fold molar excess of Xyr1 resulted in a complete shift of this probe, thereby suggesting an interaction of Xyr1 with XRE (Fig. 3A). The same molar excess of BSA was applied as a control and did not lead to a shift of the probe (Fig. 3B, lane 6). Furthermore, the specificity of the binding by Xyr1 was validated in a competition experiment. Increasing amounts of an unlabelled probe stepwise reduced and finally completely abolished the shift of the probe (Fig. 3B, lanes 2–5). Thus, the newly identified XRE is a further recognition site for Xyr1. To characterize the impact of XRE as an alternative binding site to XBS, the preference of Xyr1 for either of these DNA-binding motifs was investigated in more detail.

### Xyr1 prefers binding to XBS compared to XRE

**Figure 4.**
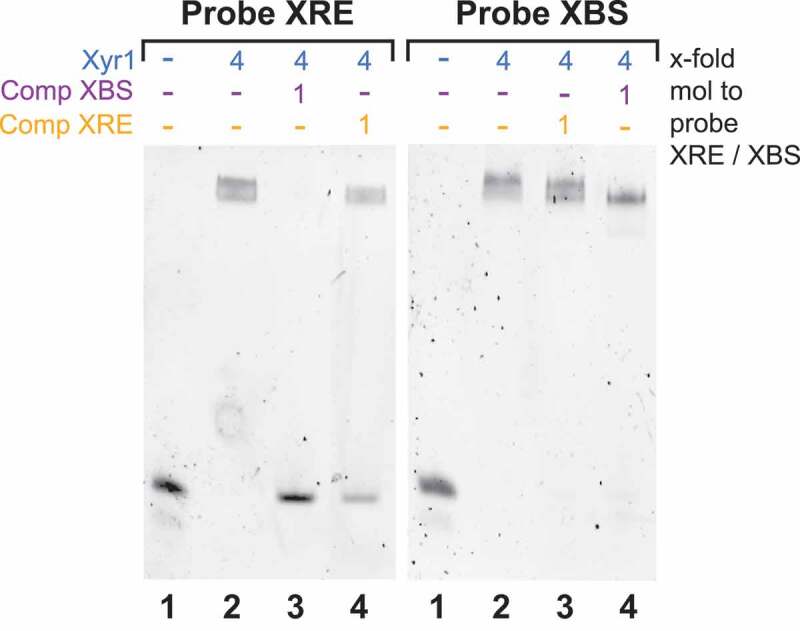
Comparative analysis of the binding of Xyr1 to XRE and XBS. EMSAs using the FAM-labelled probe containing the XRE (Probe XRE) or the FAM-labelled probe containing XBS (Probe XBS) together with Xyr1, the unlabelled probe containing XBS (Comp XBS) or the unlabelled probe containing the XRE (Comp XRE). A total of 33.4 ng of the FAM-labelled probe alone (lanes 1) or together with a 4-fold molar excess of Xyr1 (lanes 2) or together with a 4-fold molar excess of Xyr1 and equimolar amounts of either of the unlabelled probes (lanes 3 and 4) were applied.

For the abovementioned reason, we performed comparative EMSAs using the probe described above and, as a second probe, a part of a promoter sequence of a Xyr1 target gene (i.e., *xyn1*), which contains two XBSs arranged as inverted repeats. In the first experiment, the FAM-labelled probe containing the XRE was used together with Xyr1 alone or Xyr1 and one of the two investigated probes without labelling. The XRE probe together with a 4-fold molar excess of Xyr1 resulted in a full shift (Fig. 4, left image, lane 2). This shift was completely lost when the unlabelled probe containing the XBS was used as competitor (Fig. 4, left image, lane 3), while it was only weakened when the same probe was added as a cold competitor (Fig. 4, left image, lane 4). In the second experiment, the FAM-labelled probe containing the XBS was used in an analogous manner. Again, the usage of a 4-fold molar excess of Xyr1 resulted in a full shift (Fig. 4, right image, lane 2). In this case, equimolar amounts of the same probe added as a cold competitor reduced the shift (Fig. 4, right image, lane 4). More importantly, the usage of the unlabelled probe containing the XRE did not weaken the shift at all (Fig. 4, right image, lane 3). These results suggest that when both binding sites are present, Xyr1 preferentially binds to XBS, which is present in the promoters of its target genes, rather than to XRE, which is present in its own promoter.

Since a higher affinity of Xyr1 for XBS compared to XRE could be postulated and a distinct regulatory impact needs to be considered, the folding of Xyr1 in the two cases was investigated. Therefore, we employed circular dichroism (CD) spectroscopy, a method suitable for studying the composition of secondary structures of a protein [29,30]. Typically, CD-analysis of Xyr1 yields a characteristic spectrum indicating a large α-helical content that appears as negative peaks of the ellipticity at 208 nm and 222 nm. The addition of either of the previously described probes decreased the negative ellipticity in this range of wavelengths (Fig. S3). This result indicates changes in the secondary structure of Xyr1 when it is bound to either of its target sites. However, the loss of mean residue ellipticity at 222 nm was more pronounced in the presence of XRE than in the presence of XBS. Obviously, a different folding of Xyr1 occurs by binding to XRE and XBS. This finding suggests that Xyr1 could fulfil different regulatory roles when it is bound to the XRE on its own promoter or to XBS in the promoters of its target genes. The findings that Xyr1 binds to XRE on its own promoter and that XRE was identified as a negative regulatory element of *xyr1* expression raised the possibility that XRE mediates a negative feedback regulation of Xyr1.

### Xyr1 expression is negatively auto-regulated

To investigate the possible impact of Xyr1 on its own expression *in vivo*, we analysed the levels of *xyr1* transcripts originating from the native locus in a strain overexpressing Xyr1 in *trans*. For this purpose, strain TX(WT) was constructed. This strain expresses *xyr1* under the strong constitutive *tef1* promoter, while the native *xyr1* locus bears a non-sense point mutation leading to a stop-codon at position aa 81 of Xyr1 (Derntl, Mach and Mach-Aigner, unpublished data). Consequently, the use of suitable primers allows the exclusive detection of *xyr1* transcript levels produced from the native locus (hereafter referred to as *xyr1ʹ*) besides the overexpression of Xyr1 from a different locus.

Strain TX(WT) and its parent strain Xyr1ʹ(81), which bears only the mutated *xyr1* gene (*xyr1ʹ*), were cultivated on different carbon sources for 24 h (glucose, glycerol, xylan) or 48 h (CMC). Total RNA was extracted from fungal mycelia, and mutant *xyr1ʹ* transcript levels were determined by reverse transcription quantitative PCR (RT-qPCR). A clear reduction of *xyr1ʹ* transcript levels in the Xyr1 overexpression strain TX(WT) compared to Xyr1ʹ(81) was observed for all investigated conditions (Table 1). Clearly, the overexpression of Xyr1 *in trans* leads to reduced *xyr1ʹ* transcript levels, which demonstrates a negative auto-regulation of Xyr1 expression.

**Table 1.** Transcript levels of *xyr1ʹ* in the absence (strain Xyr1ʹ(81)) or presence (strain TX(WT)) of the wild-type *xyr1* overexpressed in *trans.*

Carbon source	Relative transcript levels (%) *	
	Xyr1ʹ(81)	TX(WT)
Glucose	100 ± 0.0	42.5 ± 1.3
Glycerol	100 ± 7.9	57.5 ± 2.4
Xylan	100 ± 3.9	7.9 ± 0.1
CMC	100 ± 3.8	53.3 ± 10.0

*t-tests resulted in *p*< 0.05 for the cultivation on all carbon sources (details see Table S3).

### HAX1 *does not bind to either of the two types of Xyr1 dna-binding motifs*

**Figure 5.**
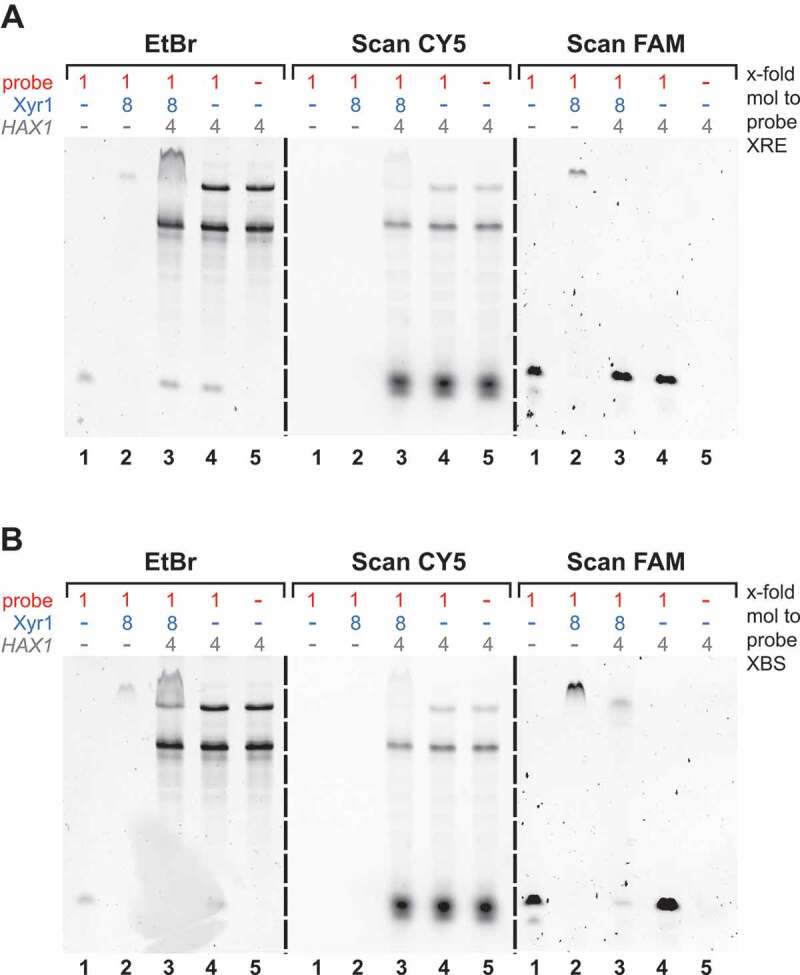
Analyses of the binding of Xyr1 and *HAX1* to XRE and XBS. EMSAs using the FAM-labelled probe containing the XRE (A) or the FAM-labelled probe containing XBS (B) alone (lanes 1) or together with Xyr1 (lanes 2), or together with *in vitro*-synthesized CY5-labelled *HAX1*_428_ (*HAX1*) (lanes 4) or together with both (lanes 3). As a control, *HAX1*_428_ was also loaded alone (lanes 5). An 8-fold molar excess of Xyr1 and a 4-fold molar excess of RNA relative to 33.4 ng of the FAM-labelled probes were used. Fluorescence and image analyses were performed via FAM-scan (right images), CY5-scan (middle images) and ethidium bromide staining (left images).

However, the relationship between the regulatory role of *HAX1* and its interaction with Xyr1 is still unclear. We investigated whether *HAX1* could compete with Xyr1 for binding to either of its DNA-binding motifs. To test the potential binding of *HAX1* to XRE in the *xyr1* promoter and to XBS in the *xyn1* promoter, EMSAs were carried out. To allow a clear distinction of the different possible interaction partners, distinctly labelled *HAX1*_428_ (CY5) and XBS- or XRE-containing probes (FAM) were employed. The EMSAs demonstrated that *HAX1* does not bind to either of the two probes (Fig. 5, all images, compare lanes 4 and 5). As observed previously, Xyr1, in contrast, leads to a shift of both probes (Fig. 5, left and right images, compare lanes 1 and 2). Therefore, a competition of *HAX1* and Xyr1 for the same DNA-binding motifs can be excluded. Interestingly, we noticed that the shift of the probe caused by the presence of Xyr1 becomes weaker when *HAX1* was additionally present in the case of the XBS-containing probe and was fully abolished in the case of the XRE-containing probe (Fig. 5, left and right images, lanes 3). This result led us to study the influence of *HAX1* on the capability of Xyr1 to bind the two types of DNA-binding motifs in detail.

### HAX1 *primarily interferes with the binding of Xyr1 to XRE*

**Figure 6.**
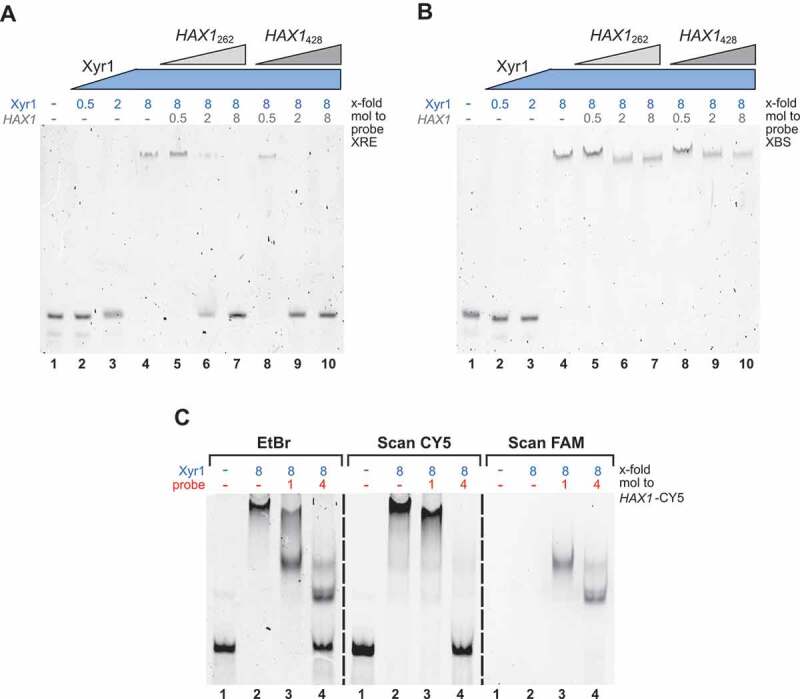
Analyses of the binding of Xyr1 to XRE and XBS in the presence of *HAX1*. EMSAs using the FAM-labelled probe containing the XRE (A) or the FAM-labelled probe containing XBS (B) alone (lanes 1) or together with different amounts of Xyr1 (lanes 2–4), or together with 8-fold molar excess of Xyr1 and increasing amounts of *HAX1*_262_ (lanes 5–7) or together with 8-fold molar excess of Xyr1 and increasing amounts of *HAX1*_428_ (lanes 8–10). (C) RNA-EMSA using 0.5 µg of *in vitro*-synthesized CY5-labelled *HAX1*_299_ (*HAX1*-CY5) alone (lane 1), or together with an 8-fold molar excess of Xyr1 (lane 2), or together with an 8-fold molar excess of Xyr1 and equimolar amounts of the FAM-labelled probe containing XBS (lane 3) or together with an 8-fold molar excess of Xyr1 and a 4-fold molar excess of the FAM-labelled probe containing XBS (lane 4). Fluorescence and image analyses were performed via FAM-scan (right image), CY5-scan (middle image) and ethidium bromide staining (left image).

Similar to the experiment described above, the two FAM-labelled probes containing either the XRE or the XBS were used. Again, an 8-fold molar excess of Xyr1 resulted in a complete shift of both probes (Fig. 6A and B, lanes 2–4). In the case of the probe containing the XRE, this shift was abolished in the presence of equimolar amounts of *HAX1* relative to Xyr1 (Fig. 6A, lanes 7 and 10). This effect could be observed independently from the *HAX1* version. However, a lower molar ratio of *HAX1*_262_ only reduced the shift, while the same concentration of *HAX1*_428_ already led to a complete loss of the shift (Fig. 6A, compare lanes 6 and 9). This provides evidence that *HAX1* interferes with the binding of Xyr1 to XRE and that *HAX1*_428_ acts as a stronger inhibitor than *HAX1*_262_. The latter is in good accordance with the fact that *HAX1*_428_ itself contains the XRE as an additional binding site for Xyr1 and is bound more efficiently by the transactivator. Therefore, this *HAX1* version can be expected to titrate Xyr1 more effectively.

In contrast, the effect of *HAX1* on the DNA-binding of Xyr1 to the XBS-containing probe is conspicuously low (Fig. 6B). While the presence of a low concentration of *HAX1* did not affect the binding of Xyr1 at all (Fig. 6B, compare lanes 4, 5, and 8), higher concentrations moved the shifted band to a slightly lower position (Fig. 6B, lanes 6, 7, 9, 10). However, in all cases when *HAX1* was present, no signal of the free probe was visible (Fig. 6B, lanes 5–10). Since the binding of *HAX1* to the XBS-containing probe was excluded earlier (Fig. 5), this finding suggests that the DNA-binding of Xyr1 to the XBS remains unchanged in the presence of *HAX1*. The altered mobility of the Xyr1-XBS complex in the presence of higher concentrations of *HAX1* might be due to less freely available Xyr1. As mentioned in the beginning, the probe contains two XBSs; thus, it can be bound by either one or two molecules of Xyr1, depending on the abundance of Xyr1. In the presence of *HAX1*, Xyr1 could be partially titrated from the probe, and the monomeric Xyr1-XBS complex would differ in its size and migration properties. Another possible explanation for the altered mobility would be a supershift resulting from the formation of a multi-component complex consisting of the probe, Xyr1 and *HAX1*.

### HAX1 *and Xyr1 do not form a multi-component complex with XBS*

To test these two possibilities, an RNA-EMSA was carried out. As described previously, a distinction of the different possible interaction partners was achieved by employing distinctly labelled *HAX1*_299_ (CY5) and XBS-containing probe (FAM). The CY5-scan of the EMSA gel validates that *HAX1* shifts in the presence of Xyr1 (Fig. 6C, middle image, compare lanes 1 and 2). When the probe is added, this shift is reduced and finally completely abolished, solely leaving the signal of unbound *HAX1* (Fig. 6C, middle image, compare lanes 2–4). Conversely, the binding of Xyr1 to the probe can be inferred from the FAM scan of the EMSA gel. Both concentrations of the probe yielded a shift arising from the formation of the Xyr1-XBS complex, but the migration properties are different (Fig. 6C, right image, lanes 3, 4). However, in both cases, the mobility of the Xyr1-XBS complex is clearly different than that of the *HAX1*-Xyr1 complex. This finding suggests binding of either *HAX1*_299_ or the XBS-containing probe to Xyr1, rather than a simultaneous binding of all three and thus the formation of a multi-component complex. Considering this finding, we suggest that the observed variation in the mobility results from the binding of either one or two molecules of Xyr1.

Finally, it should be noted that EMSAs using *HAX1*_262_ and *HAX1*_428_ yielded the same results. Hence, the latter finding can be considered to be independent of the version of *HAX1*.

### HAX1 *interferes with the negative auto-regulation of xyr1*

**Figure 7.**
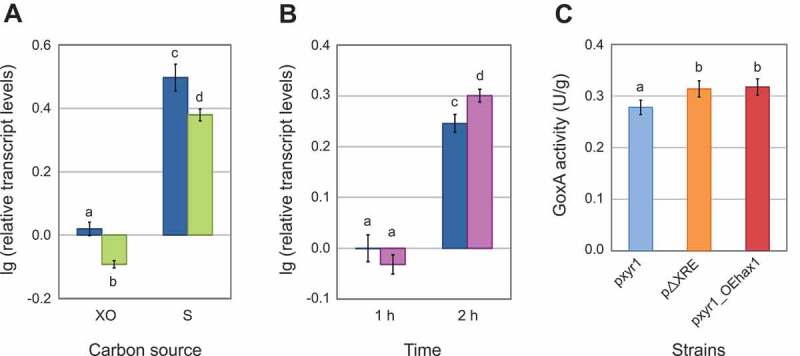
Analyses of the impact of *HAX1* on *xyr1* expression. (A) Transcript levels of *xyr1* in the presence and absence of *hax1*. The *hax1* deletion strain QM6a_Δ*hax1* (green bars) and the parent strain QM6a_loxP (blue bars) were pre-grown and transferred to minimal medium without carbon source or containing xylose (XO) or sophorose (S) for 3 h. Transcript analysis was performed in technical triplicates. Transcript levels were normalized to *act* and *sar1*, refer to cultivation without carbon source and are given in logarithmic scale (lg). Error bars indicate standard deviations; t-tests resulted in *t*(4) = −7.948, *p*< 0.01 for the cultivation on xylose and *t*(4) = −4.395, *p*< 0.05 for the cultivation on sophorose. Different letters denote significant differences among compared data. A detailed itemization of the *P* values is appended in Table S3. (B) Transcript levels of *xyr1* in the presence or absence of *hax1*_824_ overexpressed in *trans*. The *hax1* overexpression strain OE*hax1* (purple bars) and the reference strain QM6a_∆*tmus53* (blue bars) were pre-grown and transferred to minimal medium containing sophorose for 1 h and 2 h. Transcript analysis was performed in technical triplicates. Transcript levels were normalized to *act* and *sar1*, refer to QM6a_∆*tmus53* cultivated for 1 h and are given in logarithmic scale (lg). Error bars indicate standard deviations; t-tests resulted in *t*(4) = 1.672, *p*> 0.05 for the cultivation for 1 h and *t*(4) = −4.380, *p*< 0.05 for the cultivation for 2 h. Different letters denote significant differences among compared data. A detailed itemization of the *P* values is appended in Table S3. (C) GoxA activities of strains expressing the reporter gene *goxA* under the control of the full-length *xyr1* promoter (pxyr1, blue bar) or the full-length *xyr1* promoter lacking XRE (pΔXRE, orange bar) and a strain expressing the reporter gene *goxA* under the control of the full-length *xyr1* promoter and overexpressing *hax1*_428_ (pxyr1_OE*hax1*, red bar). The strains were pre-grown and transferred to minimal medium containing sophorose for 2 h. GoxA assays were performed in technical triplicates. Error bars indicate standard deviations; t-tests resulted in *t*(4) = 3.239, *p*< 0.05 for the analysis of pxyr1_OE*hax1* versus pxyr1 and *t*(4) = 0.292, *p*> 0.05 for the analysis of pxyr1_OE*hax1* versus p∆XRE. Different letters denote significant differences among compared data. A detailed itemization of the *P* values is appended in Table S3.

The fact that *HAX1* affects the binding of Xyr1 to the XRE raises the possibility that *HAX1* could interfere with the negative auto-regulation of *xyr1* expression. To test this hypothesis *in vivo*, the formation of *xyr1* transcript levels was analysed in a *hax1*-deleted background. To this end, a *hax1* deletion strain (QM6a_Δ*hax1*) was generated by applying a Cre/loxP-system [31]. This strain and its parent strain (QM6a_loxP) were incubated under different conditions (i.e., availability of carbon sources). The RNAs obtained from fungal mycelia were used as a template for RT-qPCR. Under both conditions, the transcript levels of *xyr1* were significantly lower in the *hax1* deletion strain compared to its parent strain (Fig. 7A). Similarly, *xyr1* transcript levels in a strain overexpressing *hax1* (OE*hax1*) were analysed by RT-qPCR. No significant difference in *xyr1* transcript levels of OE*hax1* and the reference strain (QM6a_Δ*tmus53*) was observed after incubation for 1 h (Fig. 7B). However, after incubation for 2 h, *xyr1* transcript levels were significantly increased in the *hax1* overexpression strain compared to the reference strain (Fig. 7B). Clearly, the presence of *HAX1* promotes *xyr1* expression, while its absence reduces *xyr1* expression. This result strengthens the assumption that *HAX1* positively affects *xyr1* transcription by acting on the negative feedback regulation of Xyr1.

For additional *in vivo* evidence of the above stated assumption, a reporter gene analysis of a p*xyr1::goxA* strain overexpressing *hax1* was performed. The p*xyr1::goxA* strain overexpressing *hax1*, i.e., pxyr1_OE*hax1*, was generated by integration of the *hax1* overexpression cassette at the *asl1* locus and the p*xyr1::goxA* cassette at the *pyr4* locus in a Δ*asl1* Δ*pyr4* double-auxotrophic mutant strain. The obtained strain pxyr1_OE*hax1* and the previously described strains pxyr1 and pΔXRE were incubated under inducing conditions, and GoxA assays were performed. The GoxA activities of pxyr1_OE*hax1* were significantly higher compared to the reference strain pxyr1, which produces standard transcript levels of *hax1* (Fig. 7C). Interestingly, the overexpression of *hax1* (strain pxyr1_OE*hax1)* and the deletion of the auto-regulatory element XRE (strain pΔXRE) have equal enhancing effects on GoxA activity (Fig. 7C). This result strongly supports the above-suggested regulatory function of *HAX1*.

**Figure 8.**
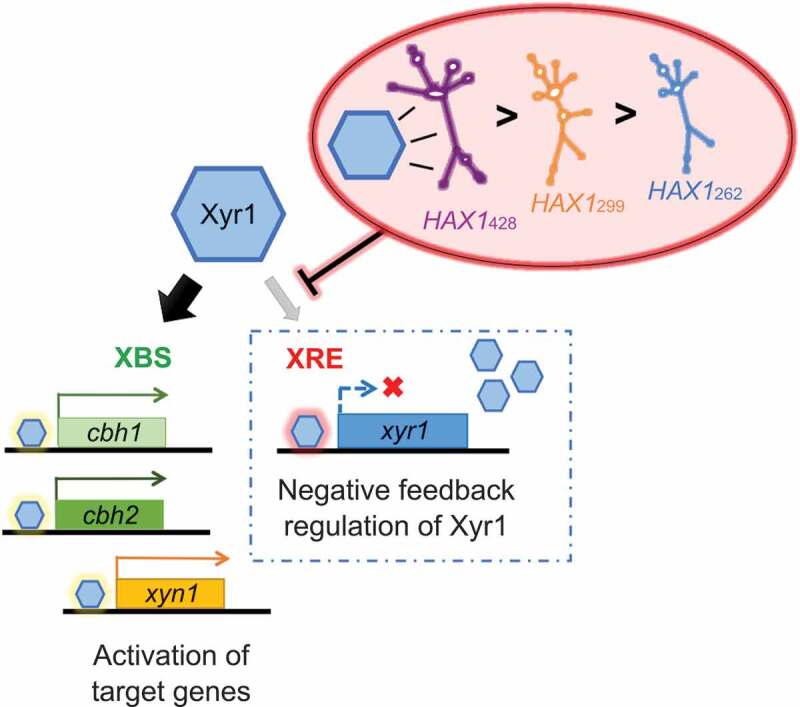
Schematic illustration of the proposed regulatory model of *HAX1* and Xyr1. Xyr1 (blue hexagon) can bind to two DNA motifs: XBS and XRE. Preferentially, Xyr1 binds to XBS at the promoter regions of its target genes (e.g., *cbh1, cbh2, xyn1*), thereby acting as a transcriptional activator (left section). When high amounts of Xyr1 are also available, the XRE on the *xyr1* promoter gets bound, thereby resulting in a negative feedback regulation of *xyr1* expression (right section). Via formation of an RNA-protein complex, *HAX1* interferes with the negative feedback regulatory loop of Xyr1, rather than with its function as an activator. Thus, *HAX1* finally has an enhancing effect on the expression of the Xyr1 target genes. Due to a higher affinity of Xyr1 for the longer versions of *HAX1, HAX1*_428_ has the largest impact on PBDE expression, and *HAX1*_299_ has a greater effect than the shortest version, *HAX1*_262._

Taken together, the results presented in this study reveal that *HAX1* interferes with the negative feedback regulatory loop of Xyr1 by impairing its binding to the XRE on its own promoter. An illustration of the supposed regulatory model and the action of *HAX1* on Xyr1 is given in Fig. 8 and discussed below.

## Discussion

In general, negative feedback regulation is a common strategy to ensure that a process stops when high amounts of the product are available. This regulation is essential to avoid unnecessary energetically demanding reactions and to maintain balance in complex metabolic pathways. However, despite the central role of Xyr1 in the regulation of PBDE expression, prior to this study, no evidence for possible auto-regulation of this transactivator had been reported.

In this study, we demonstrate that Xyr1 regulates the expression of its own gene in a negative feedback loop via binding to the currently undescribed recognition site XRE based on both *in vitro* and *in vivo* experiments. Remarkably, the XRE was found to be strikingly different in terms of its properties as a Xyr1 DNA-binding motif compared to the XBS present in the promoters of Xyr1 target genes. First, binding of Xyr1 to either of its DNA-binding motifs results in a different folding of Xyr1. Interestingly, for the close homologue of Xyr1 in *Aspergillus niger*, XlnR, the presence of a C-terminal domain mediating auto-regulation via self-masking was suggested [32]. Hence, a change in Xyr1 protein structure and, consequently, its activity in response to varying conditions (in this case binding to XBS or XRE) would not be an unusual phenomenon. Thus, Xyr1 may act as a transcriptional activator when it is bound to XBS at the regulatory regions of its target genes, whereas it blocks the expression of its own gene by binding to the XRE in the *xyr1* promoter. Second, and even more importantly, we demonstrated that Xyr1 has different affinities for XRE and XBS. EMSA studies using probes containing XBS or XRE in a competing context revealed that Xyr1 prefers binding to XBS when both DNA-binding motifs are available. This strategy of varying preferences for distinct binding sites might be a key property for the regulatory function of Xyr1. In a first instance, Xyr1 binds to classical XBS at the promoter regions of its target genes, acting as a transactivator. However, when high amounts of Xyr1 have been produced and XBS are saturated, Xyr1 also binds to XRE at the *xyr1* promoter and acts as a repressor of its own expression (Fig. 8).

However, not only Xyr1 itself but also the lncRNA *HAX1* influences the regulation of *xyr1* expression. Previously, evidence for a positive impact of *HAX1* on PBDE expression was provided; however, at that point, the regulatory strategy behind this activating function was unknown [3]. *HAX1* does not bind to XBS or XRE, but it bears several XBS, enabling physical interaction with Xyr1. Thus, the lncRNA can interfere with the binding of Xyr1 to its DNA-binding motifs on the *T. reesei* genome. Our studies revealed that *HAX1* has a greater impact on the interaction of Xyr1 with XRE in the *xyr1* promoter than on the interaction with XBS. This difference might be explained by the structural differences observed for Xyr1 when it is bound to XRE or XBS. The secondary structure of Xyr1 when the RNA-protein complex is formed might hinder the interaction of Xyr1 with XRE, rather than with XBS. Thus, *HAX1* could finally act as an activator of gene expression by dampening the negative auto-regulatory input of Xyr1. Another hypothesis is that *HAX1* predominantly reduces the amount of freely available Xyr1 when high amounts of this transactivator have been produced. In this case, the lncRNA is believed to act as a switch to shift the balance towards the activating function of Xyr1 versus negative feedback regulation. In any case, an enhancing effect of *HAX1* on *xyr1* expression could be clearly demonstrated *in vivo* by deletion and overexpression of *hax1*.

Another interesting aspect regarding the regulatory strategy of *HAX1* is the role of the different lengths of the variants of this lncRNA. It was reported that *HAX1*_262_, *HAX1*_299_ and *HAX1*_428_ have different impacts on PBDE expression [3]. During the present study, the need for different molar ratios for complex formation with Xyr1 was observed, and different inhibitory effects on the binding of Xyr1 to XRE were found. Interestingly, the longest version of *HAX1*, i.e., *HAX1*_428_, also bears XRE as a potential Xyr1 binding site. As the overexpression of this version of *HAX1* leads to the highest increase in PBDE expression [3], a functional role of this regulatory motif present at the lncRNA was suggested. However, interaction with Xyr1 is observed for all versions of *HAX1*, regardless of the presence of the XRE. Hence, it can be concluded that the XBS in the *HAX1* sequence seem to be sufficient for complex formation with Xyr1. The different effects of the *HAX1* versions on PBDE expression might also be explained by their varying lengths and properties. The presented data raise the assumption that Xyr1 has the highest affinity for long versions of *HAX1*; hence, *HAX1*_428_ is believed to have a greater impact of the negative feedback regulation of Xyr1 than shorter *HAX1* versions and, finally, the strongest effect on PBDE expression.

It should be noted that XRE is not only present on *HAX1*_428_ but also at the *hax1* locus on the fungal genome. Additionally, XBS are present on the genomic locus of *hax1*. A reversed regulatory situation might be speculated: Xyr1 could be targeted to the *hax1* genomic locus and cause a specific regulation of the expression of longer or shorter *hax1* versions and might be involved in transcription control. Tight regulation of the ratio of the *HAX1* versions is likely to be a crucial aspect in the complex interplay of *HAX1* and Xyr1. Further studies are required to gain detailed knowledge on the function of XRE on *HAX1*_428_ and the corresponding gene locus.

In summary, the finding that *HAX1* mainly affects the binding of Xyr1 to XRE suggests that *HAX1* interferes with the auto-regulatory loop of Xyr1 but not with transcriptional activation at the promoter regions of Xyr1 target genes and thus has an overall stimulating effect on gene expression (Fig. 8). The postulated concept is consistent with the generated *in vivo* data. Taken together, the results of this study regarding the negative feedback regulation of Xyr1 and the role of *HAX1* provide an interesting model for a sophisticated strategy of the two regulators and provides a first step in understanding a hitherto completely undescribed mechanism of gene regulation in fungi.

## Materials and methods

### Fungal strains and growth conditions

All strains used in this study are listed in Table S1. Strains resulting from reconstitution of *pyr4*, i.e., pxyr1, p804, p606, p497, p372, pΔXRE, OE*hax1* and pxyr1_OE*hax1* were grown on Mandels-Andreotti (MA) medium [33] containing 1% (w/v) D-glucose as the sole carbon source at 30°C. The uridine auxotrophic strain OE*hax1*_∆*pyr4* resulting from reconstitution of *asl1* was maintained on MA medium containing 1% (w/v) D-glucose, 0.1% (w/v) peptone and 5 mM uridine. Any other strains were maintained on malt extract (MEX) agar at 30°C. If applicable, uridine, L-arginine and hygromycin B were added to final concentrations of 5 mM, 2.5 mM and 113 U/ml, respectively.

For carbon source replacement experiments, mycelia were pre-cultured in 200 ml of MA medium supplemented with 0.1% peptone and 1% (w/v) glycerol as the sole carbon source on a rotary shaker (180 rpm) at 30°C for 24 h. A total of 10^9^ conidia per litre (final concentration) were used as the inoculum. For the *xyr1* promoter deletion analysis by a *goxA* reporter gene assay, pre-grown mycelia were washed, and equal amounts were resuspended in 20 ml MA medium without carbon source or MA medium containing 1.5 mM sophorose. Samples were taken after 8 h of incubation from two (p804, p606, p497, p372 and pΔXRE) or three (pxyr1) biological replicates derived from independently generated strains and analysed in technical triplicates. For the *xyr1* auto-regulation analysis by a *goxA* reporter gene assay, pre-grown mycelia of strains pxyr1, pΔXRE and pxyr1_OE*hax1* were washed, and equal amounts were resuspended in 20 ml MA medium containing 1.5 mM sophorose. Samples were taken after 2 h of incubation and analysed in technical triplicates. For transcript analysis in strain QM6a_Δ*hax1*, pre-grown mycelia were washed, and equal amounts were resuspended in 20 ml MA medium without carbon source and MA medium containing 1.5 mM sophorose or 0.5 mM D-xylose. Samples were taken after 3 h and analysed in technical triplicates. For transcript analysis in strain OE*hax1*, pre-grown mycelia were washed, and equal amounts were resuspended in 20 ml MA medium containing 1.5 mM sophorose. Cultures were harvested after 1 h or 2 h from separate cultivations, and samples were analysed in technical triplicates.

For transcript analysis in strains Xyr1ʹ(81) and TX(WT), direct cultivations were performed in 50 ml MA medium containing 1% (w/v) glucose, glycerol, xylan from beechwood (Carl Roth GmbH + Co KG, Karlsruhe, Germany) or CMC on a rotary shaker (180 rpm) at 30°C. A total of 10^9^ conidia per litre (final concentration) were used as the inoculum. Samples from biological triplicates were taken after 24 h (glucose, glycerol, xylan, lactose) or 48 h (CMC), pooled for each condition, and analysed in technical duplicates.

### Plasmid construction

For the construction of the plasmids pCD-Rpyr4/pxyr1::goxA, pCD-Rpyr4/pxyr1_804::goxA, pCD-Rpyr4/pxyr1_606::goxA, pCD-Rpyr4/pxyr1_497::goxA, pCD-Rpyr4/pxyr1_372::goxA and pCD-Rpyr4/pxyr1_ΔXRE::goxA, the different lengths of the *xyr1* promoter were PCR amplified from chromosomal DNA of *T. reesei* QM6a using the reverse primer pxyr1_rv_bam-nhe and the respective forward primer pxyr1_fw_cfr (pxyr1) or pxyr1_fw_804_cfr (p804) or pxyr1_fw_606_cfr (p606) pxyr1_fw_497_cfr (p497) or pxyr1_fw_372_cfr (p372). The *xyr1* promoter fragment lacking the XRE was generated via splicing by overlap-extension (SOE) PCR. First, two overlapping fragments were amplified from the chromosomal DNA of *T. reesei* QM6a using the primers pxyr1_fw_cfr and pxyr1_Δpal_rv (fragment 1) or pxyr1_Δpal_fw and pxyr1_rv_bam-nhe (fragment 2). Then, fragments 1 and 2 were used as templates for the SOE PCR with the primers pxyr1_fw_cfr and pxyr1_rv_bam-nhe, and the final product was extracted from a gel. All *xyr1* promoter variants were purified and blunt-end ligated into pJET1.2 (Thermo Scientific, Waltham, MA, USA), yielding the respective pJET-p*xyr1* plasmids. The *goxA* gene from *A. niger* was PCR amplified from pLW-WT [34] using the primers goxa_fw_bam and goxa_rv_bcu-nhe, also blunt-end ligated into pJET1.2 and released by digestion with BamHI and NheI. Subsequently, the resulting *goxA* fragment was inserted into the different pJET-p*xyr1* plasmids digested with the same enzymes. Finally, the complete promoter-reporter constructs were excised using BcuI and Cfr9I and ligated into a BcuI and Kpn2I-digested pCD-RPyr4T [26] vector, yielding the final plasmids.

For the construction of the plasmid pJET_Δhax1_5ʹ-hph-3ʹ, the 5ʹ and 3ʹ regions flanking *hax1*_428_ were PCR amplified from chromosomal DNA of *T. reesei* QM6a. LoxP sites were attached using the primers 5-Dhax for and 5-Dhax rev_LoxP-XmaJI (5ʹ flank) or 3-Dhax for_LoxP-XbaI-Acc65I and 3-Dhax rev_NcoI (3ʹ flank). In the first step, the 1019-bp 5ʹ flank fragment was extracted from a gel and blunt-end ligated into pJET1.2 (Thermo Scientific). The appropriate orientation was verified by digestion with XmaJI and XbaI or XmaJI and Kpn2I. Subsequently, the 1058-bp 3ʹ flank fragment was digested with XbaI and NcoI, extracted from a gel, and cloned into the pJET-5ʹ flank vector digested with XbaI and XmaJI. Finally, the *hph* gene (encoding the hygromycin B phosphotransferase) was PCR-amplified from the vector pRLMex_30_ [35] using the primers HygR for_XmaJI and HygR rev_Acc65I. The 2540-bp PCR product was digested with Acc65I and XmaJI, extracted from a gel and inserted into the pJET-5ʹ/3ʹ flank vector digested with the same enzymes, thereby interrupting the 3ʹ and 5ʹ flanking regions of *hax1*. The final plasmid was verified by sequencing (Microsynth, Balgach, Switzerland).

For the construction of the plasmid pCD-RPyr4T/OE*hax1*_428_, the 997-bp *bgl1* promoter was PCR-amplified using the primers Pbgl1 for_Kpn2I and Pbgl rev_XbaI and chromosomal DNA of *T. reesei* QM6a as a template. The purified PCR product was blunt-end ligated into pJET1.2 (Thermo Scientific), and the appropriate orientation was verified by digestion with XbaI. In a next step, *hax1*_428_ was amplified from the chromosomal DNA of *T. reesei* QM6a using the primers for_RutC30_XbaI and rev_3ʹQM6a_BcuI-NcoI. The PCR-amplified *hax1* DNA fragment was purified, digested with XbaI and NcoI, and subsequently cloned into pJET-p*bgl1* digested with XbaI and NcoI. For construction of the final *hax1* overexpression cassette, the p*bgl1-hax1* fusion product was isolated from the plasmids by digestion with Kpn2I and BcuI, extracted from a gel, and introduced into BcuI/Kpn2I-digested pCD-RPyr4T carrying the *cbh2* terminator [26] in forward orientation. The final plasmid was verified by sequencing (Microsynth).

For the construction of the plasmid pCD-RAsl1/OE*hax1*_428_, the cassette p*bgl1::hax1*::t*cbh2* was PCR amplified from pCD-RPyr4T/OE*hax1*_428_ plasmid DNA using the primers Pbgl1 for_Kpn2I and Tcbh2_rev_NheI. The 2391-bp DNA fragment was extracted from a gel, digested with Kpn2I and NheI, purified, and introduced into Kpn2I/NheI-digested pCD-RAsl1 [26] in reverse orientation. The final plasmid was verified by sequencing (Microsynth).

For the construction of pUC18-PT7-*hax1* plasmids for RNA *in vitro* synthesis, the T7-promoter was attached to the *hax1* genes (i.e., *hax1*_262_, *hax1*_299_ and *hax1*_428_) via PCR with the primer pairs hax1 for_QM6a_PT7_HindIII and hax1 rev_3ʹQM6a_XbaI (*hax1*_262_) or hax1 for_QM9414_PT7_HindIII and hax1 rev_3ʹQM6a_XbaI (*hax1*_299_) or hax1 for_RutC30_PT7_HindIII and hax1 rev_3ʹQM6a_XbaI (*hax1*_428_). As a template, chromosomal DNA of *T. reesei* was used. Both PT7-*hax1* and pUC18 were digested with HindIII and XbaI, purified and ligated.

*Escherichia coli* strain Top10 (Invitrogen, Life Technologies, Paisley, UK) was used for all cloning procedures. This strain was maintained on LB supplemented with 100 µg/ml ampicillin or spectinomycin and grown at 37°C. All PCRs were performed by applying peqGOLD Pwo DNA polymerase (PEQLAB, Biotechnologie, Erlangen, Germany) according to the manufacturer’s instructions. The primers used are listed in Table S2.

### Fungal transformation

Protoplast transformation of *T. reesei* was performed as described previously [36].

For the generation of the strains pxyr1, p804, p606, p497, p372 and pΔXRE, 80 μg of NotI-digested DNA of plasmid pCD-Rpyr4/pxyr1::goxA, pCD-Rpyr4/pxyr1_804::goxA, pCD-Rpyr4/pxyr1_606::goxA, pCD-Rpyr4/pxyr1_497::goxA, pCD-Rpyr4/pxyr1_372::goxA or pCD-Rpyr4/pxyr1_ΔXRE::goxA (precipitated and resolved in 15 μl sterile, distilled H_2_O) was used for the transformation of 10^7^ protoplasts (in 200 μl) of QM6a_∆*tmus53*_∆*pyr4*. For selection of uridine prototrophy, 500 μl of the transformation reaction mixture was added to 20 ml melted, 50°C warm MA medium agar containing 1.2 M sorbitol and 1% (w/v) D-glucose. This mixture was poured into sterile petri dishes. After solidification, the plates were incubated at 30°C for 3 to 7 days until colonies were visible.

Strain pxyr1_OE*hax1* was generated in two steps. In the first step, the *hax1* overexpression construct was integrated at the *asl1* locus of QM6a_∆*tmus53*_∆*pyr4*_ ∆*asl1*, yielding the arginine prototrophic strain OE*hax1*_∆*pyr4*. In a second step, the p*xyr1::goxA* construct was integrated at the *pyr4* locus of OE*hax1*_∆*pyr4*. For the generation of OE*hax1*_∆*pyr4*, 40 μg of NotI-digested pCD-RAsl1/OE*hax1*_428_ plasmid DNA (precipitated and resolved in 15 μl sterile distilled H_2_O) was used for the transformation of 10^7^ protoplasts (in 150 μl) of QM6a_∆*tmus53*_∆*pyr4*_∆*asl1*. For selection of arginine prototrophy, 500 μl of the transformation reaction mixture was plated with 10 ml melted, 50°C warm MA medium agar containing 1.2 M sorbitol, 1% (w/v) D-glucose, 5 mM uridine and 0.025% (w/v) peptone (as an alternative to the peptone, 0.25 mM L-arginine might be added). This mixture was poured into sterile petri dishes. After solidification, the plates were incubated at 30°C for 8 days until colonies were visible. Homokaryotic OE*hax1*_∆*pyr4* strains were generated by vegetative spore propagation and verified by PCR and Southern blot analysis, as described in the section ‘Genomic characterization’. One of the homokaryotic OE*hax1*_∆*pyr4* strains was used for the generation of the pxyr1_OE*hax1* strain. 40 μg of NotI-digested pCD-Rpyr4/pxyr1::goxA plasmid DNA (precipitated and resolved in 15 μl sterile, distilled H_2_O) were used for the transformation of 10^7^ protoplasts (in 150 μl) of OE*hax1*_∆*pyr4*. For the selection of uridine prototrophy, 500 μl of the transformation reaction mixture was plated with 10 ml melted, warm (50°C) MA medium agar containing 1.2 M sorbitol and 1% (w/v) D-glucose (without peptone). This mixture was poured into sterile petri dishes. After solidification, the plates were incubated at 30°C for 4–6 days until colonies were visible.

For the generation of strain OE*hax1*, 50 µg of the NotI-digested pCD-RPyr4T/OE*hax1*_428_ plasmid DNA was used for transformation of 10^7^ protoplasts (in 150 μl) of QM6a_∆*tmus53_*∆*pyr4*. Selection for uridine prototrophy was performed as described for the generation of pxyr1_OE*hax1*.

For the generation of QM6a_Δ*hax1*, the *hax1* deletion cassette was PCR amplified from pJET_Δhax1_5ʹ-hph-3ʹ using the primers 5-Dhax for and 3-Dhax rev_NcoI and gel-purified. The obtained 25 µg DNA was used for transformation of 10^7^ protoplasts (in 150 μl) of QM6a_loxP [31]. For selection on the resistance against hygromycin B, 500 μl of the transformation reaction mixture was plated with 10 ml MEX agar containing 1.2 M sorbitol, regenerated at 30°C for 4 h, and overlaid with 10 ml medium supplemented with 200 µg/ml of hygromycin B (yielding a final concentration of 100 µg/ml per plate). After solidification, the plates were incubated at 30°C for 7 days until colonies were visible.

### Genomic characterization

Homokaryotic pxyr1, p804, p606, p497, p372 and pΔXRE strains were generated by three rounds of vegetative spore propagation on selection medium. In the case of the pxyr1 strain, the integration of the construct was tested by PCR using the primer pairs 5pyr4_fwd3 and pxyr1_rv_bam-nhe (5ʹ flank of the *pyr4* locus, 2621 bp), goxa_fwd_Bam and goxa_rv_Bcu-Nhe (*goxA* gene, 1836 bp) or tpyr4_rev2 and pyr4_3fwd (3ʹ flank of the *pyr4* locus, 1859 bp) and via Southern blot analysis. For the p804, p606, p497 and p372 strains, the integration of the construct was tested by PCR using the primer pairs 5pyr4_fwd3 and pxyr1_rv_bam-nhe (5ʹ flank of the *pyr4* locus, 2621 bp), or goxa_fwd_Bam and goxa_rv_Bcu-Nhe (*goxA* gene, 1836 bp). The strain pΔXRE was tested by PCR using the same primers and via Southern blot analysis. For Southern blot analysis, 15 µg SacII digested chromosomal DNA and a biotinylated *goxA*-specific probe for hybridization were used, yielding a signal at 1484 bp (all strains) and locus-specific fragments of 3511 bp (pxyr1 and pΔXRE), 3282 bp (p804), 3084 bp (p606), 2975 bp (p497) and 2850 bp (p372) in size.

A homokaryotic OE*hax1*_∆*pyr4* strain was generated by three rounds of vegetative spore propagation on selection medium or MEX agar containing 0.1% IGEPAL CA-630 (Sigma Aldrich, St. Louis, MO, USA). This strain was tested by PCR using the primers ArgH-2.1kf and hax1 for_QM6a_BcuI (locus-specific PCR, 2102 bp) or ArgH-2.1kf and Ppki rev-NheI (∆*asl1-*specific PCR, 1492 bp) and via Southern blot analysis. For Southern blot analysis, 15 µg BglII digested chromosomal DNA and a biotinylated 3ʹ *asl1* locus-specific probe were used, yielding a signal at 7737 bp specific for the parent strain QM6a_∆*tmus53_*∆*pyr4_*∆*asl1* and a 2029 bp fragment specific for overexpression of *hax1*.

A homokaryotic pxyr1_OE*hax1* strain was generated by one round of vegetative spore propagation on MEX agar containing 0.1% IGEPAL CA-630. This strain as tested by PCR using the primers 5pyr4_fwd3 and pxyr1_rv_bam-nhe (locus-specific PCR, 2558 bp) or 5pyr4_fwd2 and Tpyr4_rev-NotI (∆*pyr4-*specific PCR, 1500 bp) and via Southern blot analysis. For Southern blot analysis, 15 µg NcoI digested chromosomal DNA and a biotinylated 3ʹ *pyr4* locus-specific probe were used, yielding a signal at 3551 bp specific for the parent strain OE*hax1_*∆*pyr4* and a 2248 bp fragment specific for overexpression of *hax1*.

A homokaryotic OE*hax1* strain was generated by one round of vegetative spore propagation on MEX agar containing 0.1% IGEPAL CA-630 (Sigma Aldrich, St. Louis, MO, USA). The strain was tested by PCR using the primers 5pyr4_fwd3 and Pbgl rev_XbaI (locus-specific PCR, 2590 bp) or 5pyr4_fwd2 and Tpyr4_rev-NotI (∆*pyr4-*specific PCR, 1500 bp) and via Southern blot analysis. For Southern blot analysis, 30 µg NcoI digested chromosomal DNA and a biotinylated 3ʹ *pyr4* locus-specific probe were used, yielding a signal at 3551 bp specific for the parent strain QM6a_∆*tmus53_*∆*pyr4* and a 2248 bp fragment specific for overexpression of *hax1*.

A homokaryotic QM6a_Δ*hax1* strain was generated by six rounds of vegetative spore propagation on selection medium. The integration of the *hax1* deletion cassette was tested by PCR using the primer pairs 5-Dhax for and hph 5ʹ rev (5ʹ flank to *hph*, 2447 bp), HygR for_XmaJI and hax1 rev kurz (*hph* to 3ʹ flank, 2783 bp), Dhax_locus 5-up for and HygR 5-rev (locus, 1083 bp) or 5-Dhax for and hax1 rev kurz (intact *hax1*: 1606 bp, Δ*hax1*: 3798 bp). In addition, the strain was tested via Southern blot analysis. 30 µg of NcoI-digested chromosomal DNA and a biotinylated *hax1* 5ʹ locus-specific probe were used, yielding a signal for the parent strain QM6a_loxP at 1484 bp and a locus-specific signal for Δ*hax1*_loxP at 1212 bp.

For all PCRs, GoTaq G2 polymerase (Promega, Madison, WI, USA) was applied according to the manufacturer’s instructions. The primers used are listed in Table S2. Southern blot analysis was performed as described previously [26,33]. Extraction of chromosomal DNA was performed as described by Gruber and co-workers [36] and according to the adaptations described previously [26].

### Transcript analysis

For the analysis of *xyr1* transcripts, extraction of RNA from fungal mycelia and cDNA synthesis were performed as described previously [37]. Template cDNAs were diluted 1:20. Analysis was carried out in technical triplicates. The amplification mixture (final volume 15 μl) contained 7.5 μl 2 x iQ SYBR Green Mix (Bio-Rad, Hercules, USA), 100 nM forward and reverse primer, and 2.5 μl cDNA. The primers xyr1f and xyr1r were used for the amplification.

For the analysis of *xyr1ʹ* transcripts, extraction of RNA from fungal mycelia was performed as described previously [37]. 1 µg RNA was subjected to DNaseI treatment (Thermo Scientific) and was reverse-transcribed using LunaScript RT SuperMix (NEB) according to the manufacturer’s instructions. Template cDNAs were diluted 1:50. Analysis was carried out in technical duplicates from samples derived from pooled biological triplicates. The amplification mixture (final volume 15 μl) contained Luna Universal qPCR Master Mix (NEB) and 2 µl cDNA and was prepared according to the manufacturer’s instructions. The primers xyr1_q2f and xyr1_q2r_mut_2 were used for the amplification.

All qPCRs were performed in a Rotor-Gene Q system (Qiagen, Hilden, Germany). The following PCR protocols were followed: 3 min initial denaturation at 95°C followed by 45 cycles of 15 s at 95°C, 15 s at 60°C and 15 s at 72°C (for *xyr1, xyr1ʹ* and *act*) or 3 min initial denaturation at 95°C followed by 40 cycles of 15 s at 95°C and 120 s at 64°C (for *sar1*). Control reactions and data normalization using *sar1* and *act* as reference genes and calculations were performed as described previously [38]. The primers used are listed in Table S2.

### Expression and purification of Xyr1

*E. coli* BL21(DE3) (Promega) carrying the expression vector pTS1 was pre-grown in 5 ml LB medium supplemented with 1% (w/v) D-glucose and 50 μg/ml kanamycin at 37°C overnight and used for the inoculation of 200 ml ZYP-5052 medium. ZYP-5052 is composed of 186 ml ZY (10.8 g/l tryptone and 5.4 g/l yeast extract), 0.2 ml 1 M MgSO_4_, 4 ml 50 × 5052 (0.5% (w/v) glycerol, 0.05% (w/v) glucose, 0.2% (w/v) lactose) and 10 ml 20x NPS (0.5 M (NH_4_)_2_SO_4_, 1 M KH_2_PO_4_, and 1 M Na_2_HPO_4_). For auto-induction of Xyr1 expression, the culture was incubated for 4 h at 37°C and 250 rpm followed by 20 h at 18°C. The cells were harvested by centrifugation (10,000 x g, 10 min at 4°C) and frozen at −80°C. Cell pellets from 100 ml culture were then resuspended in 10 ml BB5-L binding buffer (0.5 M NaCl, 20 mM Tris-HCl pH 7.9, 5 mM imidazole, 1 µM pepstatin A, 2 µM leupeptin, 300 U Benzoase, 1 mM DTT, 1 mM EDTA, 1% (v/v) Triton X-100 and 1 mg/ml lysozyme) and incubation on an orbital mixer at moderate temperature for 30 min. The lysate was cleared by centrifugation (14,000 x g for 20 min at 4°C) and filtered through a 0.45-µm membrane. The protein (105 kDa) was purified from the extract using Novagen® HisBind® resin (Merck, Darmstadt, Germany), a modified wash buffer (0.5 M NaCl, 20 mM Tris-HCl, 40 mM imidazole, pH 7.9) and a modified elution buffer (0.5 M NaCl, 20 mM Tris-HCl, 200 mM imidazole, pH 7.9) according to the manufacturer’s guidelines. Depending on the final application, the buffer of the purified protein samples was exchanged either to EMSA buffer (10 mM Tricine, 50 mM NaCl, pH 7.4) or to buffer A for CD spectroscopy measurements (50 mM Tris, 200 mM NaCl, 50 mM NaH_2_PO_4_, 10% (v/v) glycerol, pH 7.5), applying PD-10 columns (GE Healthcare, Uppsala, Sweden) according to the manufacturer’s guidelines. Thereafter, the protein concentration was determined using a Bio-Rad Protein Assay (Bio-Rad).

### In vitro *synthesis of* HAX1

*In vitro* synthesis of *HAX1* was performed using the T7 High Yield RNA Synthesis Kit (New England Biolabs, Ipswich, MA, USA) according to the manufacturer’s instructions. For preparation of the templates for RNA *in vitro* synthesis, the plasmids pUC18-PT7-*hax1*_262_, pUC18-PT7-*hax1*_299_ and pUC18-PT7-*hax1*_428_ were linearized with XbaI (Thermo Scientific) downstream of the PT7-*hax1* insert, leaving 5ʹ overhangs. The template DNA was purified by phenol/chloroform extraction, precipitated with 1/10 volume of 3 M sodium acetate and two volumes of ethanol and resuspended in 40 µl nuclease-free water. Next, 1 µg template DNA was applied for standard RNA synthesis in a 20 µl reaction performed at 37°C for 2 h. Subsequently, the synthesized RNA was digested with DNaseI at 37°C for 15 min and again purified via phenol/chloroform extraction and precipitation with 1/10 volume of 3 M sodium acetate and 2 volumes of ethanol. Finally, the RNA pellet was resolved in 50 µl nuclease-free water, quantified via Nano Drop and analysed by denaturing polyacrylamide gel electrophoresis (PAGE). For the denaturing PAGE, 0.5–1 µg *HAX1* RNA supplemented with 1.5 volumes RNA loading dye (95% ultrapure formamide, 0.025% bromphenol blue, 0.025% xylene cyanol FF, 5 mM EDTA, pH 8.0) was heated at 95°C for 5 min and separated on a 5% polyacrylamide gel (19:1 acrylamide:bis-acrylamide) containing 8 M urea at 15 mAmp for 45 min, largely as described before [39].

### *CY5-labelling of* HAX1

CY5-coupled cytidine-5ʹ-phosphate-3ʹ-(aminohexyl)-phosphate (pCp-CY5, from Jena Bioscience, Jena, Germany) was linked to the 3ʹ end of *in vitro* synthesized *HAX1*_299_ RNA using T4 RNA Ligase (Thermo Scientific) based on the instructions provided but scaled up for labelling 294.67 pmol (30 µg) *HAX1* RNA with equimolar amounts of pCp-CY5 in a 30 µl ligation mixture. The reaction was performed at 4°C overnight and stopped by incubation at 70°C for 10 min. Subsequently, the CY5-labelled *HAX1* RNA was purified using Sephadex G-25 Quick Spin Columns for radiolabelled RNA purification (Roche, Basel, Switzerland) according to the manufacturer’s instructions. Before application for the EMSAs, the sample eluted from the column was again analysed by denaturing PAGE as described in the prior section.

### Electrophoretic mobility shift assay (EMSA)

Synthetic, 35-bp-long, FAM-labelled oligonucleotides (Sigma-Aldrich) were annealed with their complementary oligonucleotides by being heated at 95°C and subsequently cooled to room temperature, yielding labelled ds DNA fragments used as EMSA probes. Unlabelled ds DNA fragments used as cold competitors were prepared in the same way. The sequences of all oligonucleotides are given in Table S2. Similarly, *HAX1* RNA (unlabelled- or CY5-labelled) was denatured at 95°C for 5 min and cooled to room temperature immediately before addition to the reaction mixture to achieve correct folding.

The protein-DNA binding assay and non-denaturing PAGE were carried out based on the protocol described by Stangl and co-workers [40]. A total of 33.4 ng of the labelled ds DNA fragment was applied in a 10 µl reaction and supplemented with heterologously expressed Xyr1 or BSA (Sigma Aldrich) in appropriate molar ratios (0.1- to 8-fold molar excess of the protein). If applicable, other components (e.g., unlabelled ds DNA fragments, *HAX1* RNA or CY5-labelled *HAX1* RNA) were added in the molar ratio as specified in the figure legends. 33.4 ng (1.47 pmol) of the FAM-labelled ds DNA fragment correspond to equal amounts of the unlabelled ds DNA fragments, 154.35 ng Xyr1 (105 kDa), 97.76 ng BSA (66.5 kDa), 131.14 ng *HAX1*_262_ RNA (262 nt), 314.23 ng *HAX1*_428_ RNA (428 nt) and 149.66 ng CY5-labelled *HAX1*_299_ RNA (299 nt). All nucleic acids were mixed before adding the protein. Binding was achieved in EMSA buffer (10 mM Tricine, 50 mM NaCl, pH 7.4) by incubation at 22°C for 10 min. The samples were separated on a 5.8% native polyacrylamide gel (30:0.36 acrylamide:bis-acrylamide) containing 5.4% glycerol in 0.5-fold concentrated TBE at 4°C for 75 min at 160 volt and 35 mAmp per gel. Fluorescence and image analysis of the gels was carried out using a ChemiDoc^TM^ MP Imaging System with Image Lab^TM^ Software version 5.2 (Bio-Rad). If applicable, the EMSA gels were stained with ethidium bromide (1 µg/ml in 0.5-fold concentrated TBE) for 10 min and analysed using a Gel Doc^TM^ XR+ Imaging System with Image Lab^TM^ Software version 5.2 (Bio-Rad) after fluorescence scanning.

### RNA-EMSA

RNA-EMSAs were performed using the above-described labelled and unlabelled ds DNA fragments, *HAX1* RNA and CY5-labelled *HAX1* RNA, essentially following the procedure of the EMSA (see section above). However, the protocol was adapted for adequate separation of 262 nt to 428 nt *HAX1* RNA and performed under RNase-free conditions. *HAX1* RNA (0.5 µg or 1 µg) was applied in a 10 µl or 20 µl reaction and supplemented with heterologously expressed Xyr1 or BSA (Sigma Aldrich) in appropriate molar ratios (equimolar amounts or an up to 8-fold molar excess of the protein). If applicable, other components (e.g., labelled or unlabelled ds DNA fragments) were added in the molar ratio as specified in the figure legends. 1 µg of *HAX1*_262_ (11.21 pmol) and *HAX1*_428_ (6.86 pmol) RNA corresponds to 1180 ng and 720.3 ng Xyr1 (105 kDa), respectively. 1 µg of *HAX1*_299_ (9.82 pmol) RNA corresponds to 1030 ng Xyr1 (105 kDa), 653.03 ng BSA (66.5 kDa) and 223 ng labelled ds DNA fragment. All nucleic acids were mixed before adding the protein. Binding was achieved in EMSA buffer (10 mM Tricine, 50 mM NaCl, pH 7.4) by incubation at 22°C for 10 min. The samples were separated on a 4% native polyacrylamide gel (30:0.36 acrylamide:bis-acrylamide) in 0.5-fold concentrated TBE at 4°C for 45 min at 160 volt and 15 mAmp per gel. RNA-EMSA gels were analysed by ethidium bromide staining (1 µg/ml in 0.5-fold concentrated TBE) for 10 min and imaging using a Gel Doc^TM^ XR+ Imaging System with Image Lab^TM^ Software version 5.2 (Bio-Rad). If applicable, fluorescence scanning was performed before ethidium bromide staining using a ChemiDoc^TM^ MP Imaging System with Image Lab^TM^ Software version 5.2 (Bio-Rad).

### Circular dichroism (CD) spectroscopy

For CD spectroscopy measurements, 400 μl of a 500 nM Xyr1 solution exchanged to buffer A (50 mM Tris, 200 mM NaCl, 50 mM NaH2PO4, 10% (v/v) glycerol, pH 7.5) was used. The ds DNA fragments were prepared by annealing of the complementary oligonucleotides (Sigma-Aldrich) and added to Xyr1 to final concentrations of 1.8 µM or 3.6 µM. The sequences of all oligonucleotides are given in Table S2. Measurements were carried out in 0.2 cm SUPRASIL® quartz cells (Hellma Analytics, Müllheim, Germany) in a J-815 CD Spectrometer (Jasco, Tokyo, Japan) at 22°C. CD spectra of the proteins were collected from 260–200 nm as an average of 3 scans and baseline subtracted to exclude buffer influences. Data are presented as the mean residue ellipticity [θ] in deg cm^2^ dmol^−1^, i.e., (millidegrees x MRW)/(pathlength in mm x concentration in mg/ml), where MRW (mean residue weight) is 109.69 Da. Data were corrected by the changes in the concentration of Xyr1 resulting from volumetric variation due to the addition of the DNA fragments.

### Glucose oxidase (GoxA) assay

GoxA assays were performed as described previously [41] using ABTS (2,2ʹ-azino-di-(3ethyl-benzthiazoline sulphonate)) (Molekula Ltd., Gillingham, United Kingdom) and horseradish peroxidase (Sigma-Aldrich). GoxA activities given in units are means of technical triplicates (strains pxyr1, pΔXRE and pxyr1_OE*hax1*) or technical triplicates and two (strains p804, p606, p497, p372 and pΔXRE) or three (strain pxyr1) biological replicates derived from independently generated strains and were referred to the biomass (dry weight). One unit of enzymatic activity is defined as the amount of enzyme that oxidizes 1 µmol of D-glucose per min at pH 5.8 and 25°C.

### Statistical analysis

For statistical analyses of data, the program IBM® SPSS® Statistics Version 23.0.0.0 was used to perform either an ANOVA (P < 0.05) followed by a post-hoc Tukey multiple comparison test or a 2-tailed, 2-sample t-test.

## Data Availability

All data that support the findings of this study are included in the manuscript and supplementary information.
