# Supplementary material for: Regulation of gene expression by the action of a fungal lncRNA on a transactivator
Source: RNA Biol. 2019 Sep 13;17(1):47–61. doi: 10.1080/15476286.2019.1663618 (PMC6948969; doi:10.1080/15476286.2019.1663618)
Supplement: Supplemental Material [file krnb-17-01-1663618-s001.pdf]

Figure S1:

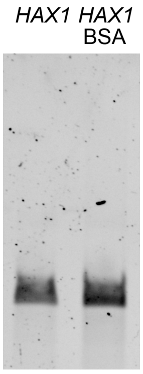

Control RNA-EMSA.

1 µg of *in vitro* synthesized $HAX1_{299}$ (*HAX1*) alone (lane 1) or together with an 8-fold molar excess of BSA (lane 2) was used.

Figure S2:

A

```
                               301                                              350
         HAX1 262      (1)  --------------------------------------------------
         HAX1 299      (1)  --------------------------------------------------
         HAX1 428      (1)  -------GAAGTTCCACACGGATACAGAGACACAACATGCAGGAGATTGG
Pxyr1_1500bp_minus   (301)  ACTGTAAGGGCGCAGAAATCGCGCCAAGCTCTGAGCAGGAAAAAGGTTGA
        Consensus    (301)         G       A A  G   CA    C  A CA G A   AG TTG
                            351                                              400
         HAX1 262      (1)  --------------------------------------------------
         HAX1 299      (1)  --------------------------------------------------
         HAX1 428     (44)  GCGTCATCCTTGTTCCACACGTTTTCTACCTAGGTAGCTGTAACA---AC
Pxyr1_1500bp_minus   (351)  GGATAGGAGCTGCTTCTGCTGCAGACTACCTAGGTAGGTATCCAGTGGAG
        Consensus    (351)  G  T      TG T C    G    CTACCTAGGTAG T T       A
                            401                                              450
         HAX1 262      (1)  --------------------------------------------------
         HAX1 299      (1)  ---------------------------------------GGTCAGGCCCG
         HAX1 428     (91)  AAGATTTACAACCAGAGCCCGAAGCTGTTCCTTGGTGATGGTCAGGCCCG
Pxyr1_1500bp_minus   (401)  GAGAGACTGATTGACTGTTCGTTAAGTCGGCATCCTTCTAAGATGAACTG
        Consensus    (401)   AGA      A   A  G CG          C T  T  TGGTCAGGCCCG
                            451                                              500
         HAX1 262      (1)  --------------------------CCCACCGGCAGGTGGCTAAACGGT
         HAX1 299     (12)  TTCAAGCCCGTTCAAGCCCGTCCAAACCCACCGGCAGGTGGCTAAACGGT
         HAX1 428    (141)  TTCAAGCCCGTTCAAGCCCGTCCAAACCCACCGGCAGGTGGCTAAACGGT
Pxyr1_1500bp_minus   (451)  CTCGGCTGGAATCAAACCGTCGCCTCTTGACGAAGATGTGACTCGCCGGC
        Consensus    (451)  TTCAAGCCCGTTCAAGCCCGTCCAAACCCACCGGCAGGTGGCTAAACGGT
                            501                                              550
         HAX1 262     (25)  GGCTGGT------GGCTGACGCCCGCGGCTTAATCAGAGGTGGGAGCTAC
         HAX1 299     (62)  GGCTGGT------GGCTGACGCCCGCGGCTTAATCAGAGGTGGGAGCTAC
         HAX1 428    (191)  GGCTGGT------GGCTGACGCCCGCGGCTTAATCAGAGGTGGGAGCTAC
Pxyr1_1500bp_minus   (501)  GACAGCTCTCACGGGCTGGGGCCGAGGGTATAA--AGAGG-GGAAAAGAG
        Consensus    (501)  GGCTGGT      GGCTGACGCCCGCGGCTTAATCAGAGGTGGGAGCTAC
                            551                                              600
         HAX1 262     (69)  TTAGCAGTCAGA-----CAAAGCCGAGATGGCGTCGAACGGCT-CATGGC
         HAX1 299    (106)  TTAGCAGTCAGA-----CAAAGCCGAGATGGCGTCGAACGGCT-CATGGC
         HAX1 428    (235)  TTAGCAGTCAGA-----CAAAGCCGAGATGGCGTCGAACGGCT-CATGGC
Pxyr1_1500bp_minus   (548)  AAAGCAGGCGAAACTCCCTATCGCGAGCGCGCCTTGACAAGCTACACGGC
        Consensus    (551)  TTAGCAGTCAGA     CAAAGCCGAGATGGCGTCGAACGGCT CATGGC
                            601                                              650
         HAX1 262    (113)  TATTGT----GGGGACGCAGCTCCTGTTC--GGCCCAGCCCGCAGGTGCT
         HAX1 299    (150)  TATTGT----GGGGACGCAGCTCCTGTTC--GGCCCAGCCCGCAGGTGCT
         HAX1 428    (279)  TATTGT----GGGGACGCAGCTCCTGTTC--GGCCCAGCCCGCAGGTGCT
Pxyr1_1500bp_minus   (598)  TGCCGGCCACGGCGACAAACCAGCAGACAATGACACAAACAG-AGAGGAT
        Consensus    (601)  TATTGT    GGGGACGCAGCTCCTGTTC  GGCCCAGCCCGCAGGTGCT
                            651                                              700
         HAX1 262    (157)  AAAACTGAATGGATGGCTGGGAGAGAAGAAGTC-GAGAACCATAAGGTGA
         HAX1 299    (194)  AAAACTGAATGGATGGCTGGGAGAGAAGAAGTC-GAGAACCATAAGGTGA
         HAX1 428    (323)  AAAACTGAATGGATGGCTGGGAGAGAAGAAGTC-GAGAACCATAAGGTGA
Pxyr1_1500bp_minus   (647)  GCAGCAGAAGAGATGGCAGGTTGGGGCAGAGACTGGGGGTTGTTAGTTAA
        Consensus    (651)  AAAACTGAATGGATGGCTGGGAGAGAAGAAGTC GAGAACCATAAGGTGA
                            701                                              750
         HAX1 262    (206)  C-GACAATACCA-AGAAGTCGGTGTATCGTAGATACTCAATGGCCAGAT-
         HAX1 299    (243)  C-GACAATACCA-AGAAGTCGGTGTATCGTAGATACTCAATGGCCAGAT-
         HAX1 428    (372)  C-GACAATACCA-AGAAGTCGGTGTATCGTAGATACTCAATGGCCAGAT-
Pxyr1_1500bp_minus   (697)  AAGACACGACCAGAGAGCTCCATC-ATGGAAGCAATATATGGGGACTCTC
        Consensus    (701)  C GACAATACCA AGAAGTCGGTGTATCGTAGATACTCAATGGCCAGAT
                            751                                              800
         HAX1 262    (253)  GAAATGCGTG----------------------------------------
         HAX1 299    (290)  GAAATGCGTG----------------------------------------
         HAX1 428    (419)  GAAATGCGTG----------------------------------------
Pxyr1_1500bp_minus   (746)  GCAATTTTTATTGCTGTTCGCTTCTCGCACCGCACCGAGGGCCTCCATCA
        Consensus    (751)  GAAATGCGTG
```

B

```
                                 2556                                              2605
    5’ end of HAX1 428      (1)  ---------------------------------------------CCTTGTT
Pxyr1-3kb_plus strand    (2556)  GTTCATCTTAGAAGGATGCCGACTTAACGAACAGTCAATCAGTCTCTCCT
                                 2606                                              2655
    5’ end of HAX1 428      (8)  CCACACGTTTTCTACCTAGGTAG-CTGTAACAACAAGATTTACA------
Pxyr1-3kb_plus strand    (2606)  CCACTGGATACCTACCTAGGTAGTCTGCAGCAGAAGCAGCTCCTATCCTC
                                 2656                                       2699
    5’ end of HAX1 428     (51)  --------------------------------------------
Pxyr1-3kb_plus strand    (2656)  AACCTTTTTCCTGCTCAGAGCTTGGCGCGATTTCTGCGCCCTTA
```

BLAST analysis of *HAX1* and the promoter sequence of *xyr1*.

(A) Result of the BLAST analysis using the *xyr1* upstream region (-1500 bp from ATG, minus strand) and the sequence of the three *HAX1* versions (i.e. *HAX1*$_{262}$, *HAX1*$_{299}$ and *HAX1*$_{428}$). **(B)** Result of the BLAST analysis using the *xyr1* upstream region (-3000 bp from ATG, plus strand) and the 50 nt sequence proximal to the 5’-end of *HAX1*$_{428}$. 50 nt per row are given. Numbers in brackets give the position of the first nucleotide in this row relative to the respective total-length sequence. Nucleotides conserved in all aligned sequences are highlighted in yellow; nucleotides conserved in some of the aligned sequences are highlighted in blue. Gaps in aligned sequences are indicated by dashes. The identified 12 bp palindromic sequence XRE is highlighted by a red frame.

Figure S3:

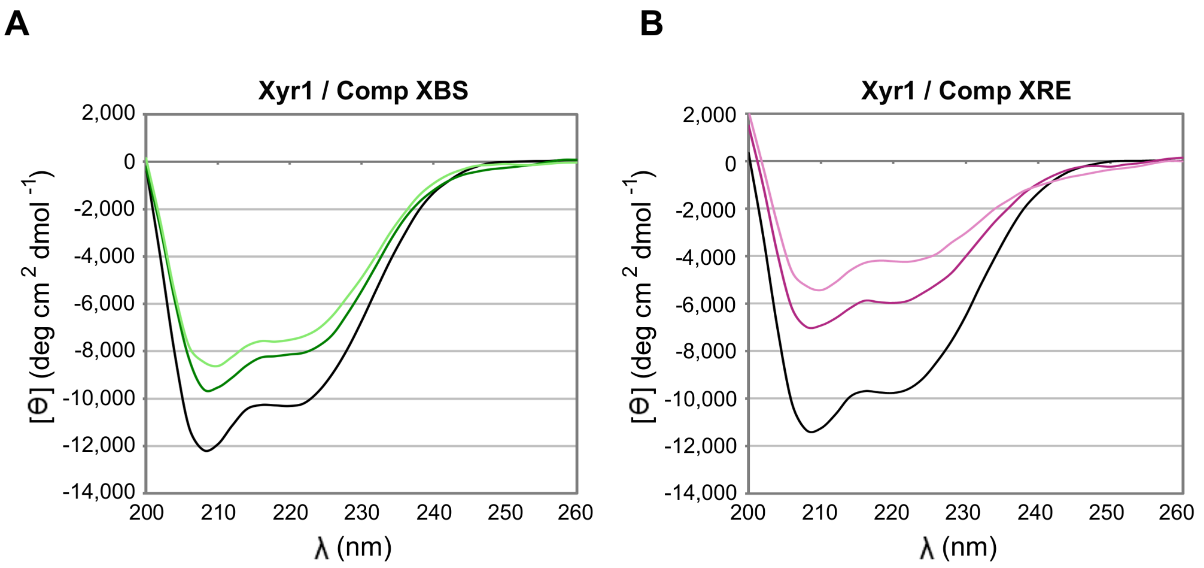

Spectra of CD analyses of Xyr1 in presence of its two types of DNA-binding motives.

(A) Far-UV spectrum (200 – 260 nm) of CD analyses using 0.5 µM Xyr1 before (black line) and after the addition of 16.85 µg (dark green) or 33.69 µg (light green) of the unlabeled EMSA probe containing XBS (Comp XBS), yielding final concentrations of 1.8 µM or 3.6 µM, respectively. **(B)** Far-UV spectrum (200 – 260 nm) of CD analyses using 0.5 µM Xyr1 before (black line) and after the addition of 16.85 µg (dark purple) or 33.69 µg (light purple) of the unlabeled EMSA probe containing the XRE (Comp XRE), yielding final concentrations of 1.8 µM or 3.6 µM, respectively.

Table S1: Fungal strains used in this study

Abbreviation	Strain background	Genetic modification	Properties	Employment	Source
-	QM6a_Δ*tmus53*_Δ*pyr4*		Wild-type like with auxotrophic marker	Transformation (recipient strain)	[26]
pxyr1	QM6a_Δ*tmus53*	p*xyr1*::*goxA*::t*cbh2*	1033 bp of *xyr1* promoter, *goxA* reporter gene, re-establishment of *pyr4*	GoxA assay	This study
p804	QM6a_Δ*tmus53*	p*xyr1*_804::*goxA*::t*cbh2*	804 bp of *xyr1* promoter, *goxA* reporter gene, re-establishment of *pyr4*	GoxA assay	This study
p606	QM6a_Δ*tmus53*	p*xyr1*_606::*goxA*::t*cbh2*	606 bp of *xyr1* promoter, *goxA* reporter gene, re-establishment of *pyr4*	GoxA assay	This study
p497	QM6a_Δ*tmus53*	p*xyr1*_497::*goxA*::t*cbh2*	497 bp of *xyr1* promoter, *goxA* reporter gene, re-establishment of *pyr4*	GoxA assay	This study
p372	QM6a_Δ*tmus53*	p*xyr1*_372::*goxA*::t*cbh2*	372 bp of *xyr1* promoter, *goxA* reporter gene, re-establishment of *pyr4*	GoxA assay	This study
pΔXRE	QM6a_Δ*tmus53*	p*xyr1*_ΔXRE::*goxA*::t*cbh2*	Deletion of XRE in *xyr1* promoter, *goxA* reporter gene, re-establishment of *pyr4*	GoxA assay	This study
Xyr1'(81)	QM6a_Δ*tmus53*_Δ*pyr4*	*xyr1*'(81)	Non-sense mutation at position 81 in native *xyr1*	RT-qPCR	Derntl, Mach and Mach-Aigner, unpublished data
TX(WT)	QM6a_Δ*tmus53*	*xyr1*'(81)<br>ptef::*xyr1*	Non-sense mutation at position 81 in native *xyr1*,<br>Overexpression of *xyr1* under *tef1* promoter, re-establishment of *pyr4*	RT-qPCR	Derntl, Mach and Mach-Aigner, unpublished data
OE*hax1*	QM6a_Δ*tmus53*	p*bgl1*::*hax1*$_{428}$::t*cbh2*	Overexpression of *hax1*, re-establishment of *pyr4*	RT-qPCR	This study

-	QM6a_Δ*tmus53*		Wild-type like	Reference strain	[31]
-	QM6a_Δ*tmus53*_Δ*pyr4*_ Δ*asl1*		Wild-type like with two auxotrophic markers	Transformation (recipient strain)	[26]
OE*hax1*_Δ*pyr4*	QM6a_Δ*tmus53*_Δ*pyr4*	p*bgl1*::*hax1*$_{428}$::t*cbh2*	Overexpression of *hax1*,<br>re-establishment of *asl1*	Transformation (recipient strain)	This study
pxyr1_OE*hax1*	QM6a_Δ*tmus53*	p*bgl1*::*hax1*$_{428}$ ::t*cbh2*<br>p*xyr1*::*goxA*::t*cbh2*	Overexpression of *hax1*,<br>re-establishment of *asl1*<br>1033 bp of *xyr1* promoter,<br>*goxA* reporter gene,<br>re-establishment of *pyr4*	GoxA assay	This study
QM6a_loxP	QM6a_Δ*tmus53*_Δ*pyr4* (loxP)		Integration of Cre recombinase at the *pyr4* locus	Transformation (recipient strain) reference strain	[31]
QM6a_Δ*hax1*	QM6a_Δ*tmus53*_Δ*pyr4* (loxP)	Δ*hax1* (loxP)	Deletion of *hax1*, HygR	RT-qPCR	This study

Table S2: Primers and probes used in this study

Name	Sequence (5' - 3')	Employment(s)
actf	TGAGAGCGGTGGTATCCACG	qPCR
actr	GGTACCACCAGACATGACAATGTTG	qPCR
ArgH-2.1kf	CACCCTTTGTAACACGAGTCG	OE*hax1*_Δ*pyr4* test-PCR
3-Dhax for_LoxP-XbaI-Acc65I	CAAATCTAGAGGTACCATAACTTCGTATAGCATACATTATACGAAGTTATTAAATGATTACATACTTCCGTACC	Δ*hax1* cloning
5-Dhax for	GCAATTACGAGGCCATCATC	Δ*hax1* cloning, test-PCR
3-Dhax rev_NcoI	CAAACCATGGGAGACCAACTCAGCGCAAAAG	Δ*hax1* cloning
5-Dhax rev_LoxP-XmaJI	CAAACCTAGGATAACTTCGTATAGCATACATTATACGAAGTTATCTGTATCCGTGTGGAACTTCTTAC	Δ*hax1* cloning
Dhax_locus 5-up for	CTACTTGCCCCTGGATCCCC	Δ*hax1* test-PCR
EMSA Pxyr1_fw Pal-FAM	[FAM]-CACTGGATACCTACCTAGGTAGTCTGCAGCAGAAG	EMSA probe XRE
EMSA Pxyr1_fw Pal	CACTGGATACCTACCTAGGTAGTCTGCAGCAGAAG	EMSA probe XRE, Circular dichroism
EMSA Pxyr1_rev Pal	CTTCTGCTGCAGACTACCTAGGTAGGTATCCAGTG	EMSA probe XRE, Circular dichroism
goxa_fw_bam	GGATCCATGCAGACTCTCCTTGTGAGCTCG	p*xyr1*::*goxA* constructs
goxa_rv_bcu-nhe	GCTAGCACTAGTTCACTGCATGGAAGCATAATCTTCC	p*xyr1*::*goxA* constructs
hax1 for_QM6a_BcuI	CAGCAGTACTAGTCCCACCGGCAGGTGGCTAAACGG	OE*hax1*_Δ*pyr4* test-PCR
hax1 for_QM6a_PT7_HindIII	CAGCAGAAGCTTTAATACGACTCACTATAGGGCCCACCGGCAGGTGGCTAAACGG	pUC18-*hax1* constructs
hax1 for_QM9414_PT7_HindIII	CAGCAGAAGCTTTAATACGACTCACTATAGGGGGTCAGGCCCGTTCAAGCCCGTTC	pUC18-*hax1* constructs
hax1 for_RutC30_PT7_HindIII	CAGCAGAAGCTTTAATACGACTCACTATAGGGGAAGTTCCACACGGATACAGAGACACAACATG	pUC18-*hax1* constructs
hax for_RutC30_XbaI	CAACTCTAGAGAAGTTCCACACG	OE*hax1* construct

	GATACAGAGACACAACATG	
hax1 rev kurz	TGAGTCGAGGGGCTACTGCAAGTAC	Δ*hax1* test-PCR
hax1 rev_3’QM6a_BcuI-NcoI	CAACCCATGGACTAGTCACGCATTTCATCTGGCCATTGAGTATCTACG	OE*hax1* construct
hax1 rev_3’QM6a_XbaI	CAGCAGTCTAGACACGCATTTCATCTGGCCATTGAGTATCTACG	pUC18-*hax1* constructs
hph 5' rev	GAAGAAGATGTTGGCGACCTCG	Δ*hax1* test-PCR
HygR for_XmaJI	CAAACCTAGGAGATAACGGTGAGACTAGCGGC	Δ*hax1* test-PCR
HygR rev_Acc65I	CAAAGGTACCGCGCTATTAACGTTTGGAAAGC	Δ*hax1* test-PCR
HygR 5-rev	CAACGTGGACAGCTGGATAAGG	Δ*hax1* test-PCR
Pbgl1 for_Kpn2I	CAAGTCCGGAGCAAGCGATAACCATAGGTA	OE*hax1* construct
PbgI rev_XbaI	CAACTCTAGACTCAACAAAGCAGAGTCTTG	OE*hax1* construct
Ppki rev-NheI	CAAGGCTAGCGTCGGGAGGGGGGGAGAGAAATC	OE*hax1_Δpyr4* test-PCR
Prxyn1.1f_FAM	[FAM]-TTGGCAGGCTAAATGCGACATCTTAGCCGGATGCA	EMSA probe XBS
Pxyn1.1f	TTGGCAGGCTAAATGCGACATCTTAGCCGGATGCA	EMSA probe XBS, Circular dichroism
Pxyn1.1r	TGCATCCGGCTAAGATGTCGCATTTAGCCTGCCAA	EMSA probe XBS, Circular dichroism
pxyr1_fw_cfr	CCCGGGCCATCTACACAAGAGCAATGGCC	p*xyr1*::*goxA* constructs
pxyr1_372_fw_cfr	CCCGGGTCTGCAGCAGAAGCAGCTCCTATCCTCAACC	p*xyr1*::*goxA* constructs
pxyr1_497_fw_cfr	CCCGGGGCCGGCGAGTCACATCTTCGTCAAGAGG	p*xyr1*::*goxA* constructs
pxyr1_606_fw_cfr	CCCGGGCCGTGGCCGGCAGCCGTGTAGCT	p*xyr1*::*goxA* constructs
pxyr1_804_fw_cfr	CCCGGGGCAAGCCTTGTCTGATGGAGGCCCTCGG	p*xyr1*::*goxA* constructs
pxyr1_rv_bam-nhe	GCTAGCATGCGGATCCTGTGGCGCGCTGTGTG	p*xyr1*::*goxA* constructs pxyr1_OE*hax1* test-PCR
pxyr1_Δpal_fw	TCTCTCCTCCACTGGATACTCTGCAGCAGAAGCAGCTCCTATCCTCAACC	p*xyr1*::*goxA* constructs

pxyr1_Δpal_rv	CTGCTTCTGCTGCAGAGTATCCAGTGGAGGAGAGACTGATTGACTGTTCG	p*xyr1*::*goxA* constructs
pyr4_3fwd	AGACGAGGACCAGCAGACC	p*xyr1*::*goxA* test-PCR
5pyr4_fwd2	CACCACAACCAGTGAAGAGCTAC	OE*hax1* test-PCR
5pyr4_fwd3	CCAGACGGTGATTCACATATACG	OE*hax1* and p*xyr1*::*goxA* test-PCR
sar1fw	TGGATCGTCAACTGGTTCTACGA	qPCR
sar1rev	GCATGTGTAGCAACGTGGTCTTT	qPCR
Tcbh2_rev_NheI	GCTAGCGCTATTAACGTTTGGAAAGC	OE*hax1*_Δ*pyr4* construct
Tpyr4_rev2	CAGGAAGCTCAGCGTCGAG	OE*hax1* and p*xyr1*::*goxA* test-PCR
xyr1f	CCCATTCGGCGGAGGATCAG	qPCR
xyr1r	CGAATTCTATACAATGGGCACATGGG	qPCR
xyr1_q2f	TCCGTCGCTATTCTGCCTAC	qPCR
xyr1_q2r_mut_2	CAGCAGTACCCGTTGAATGG	qPCR

Table S3: Statistics

Statistical analysis (ANOVA and post-hoc Tukey multiple comparison-test) of the results from the GoxA reporter gene assay in Fig 2B. $F(9;12) = 11.684$

Strain_Condition		*P* value	Significant?
p372_NCS	p372_S	1.0000	No
	p497_NCS	0.0017	Yes
	p497_S	0.0078	Yes
	p606_NCS	0.0013	Yes
	p606_S	0.0007	Yes
	p804_NCS	0.0020	Yes
	p804_S	0.0016	Yes
	pxyr1_NCS	0.0043	Yes
	pxyr1_S	0.0017	Yes
p372_S	p372_NCS	1.0000	No
	p497_NCS	0.0028	Yes
	p497_S	0.0133	Yes
	p606_NCS	0.0021	Yes
	p606_S	0.0011	Yes
	p804_NCS	0.0033	Yes
	p804_S	0.0026	Yes
	pxyr1_NCS	0.0076	Yes
	pxyr1_S	0.0029	Yes

NCS, no carbon source; S, sophorose

Statistical analysis (2-tailed, 2-sample T-test) of the results from the GoxA reporter gene assay of strains in Fig. 2C.

Strain_Condition		*t*	df	*P* value	Significant?
pΔXRE_NCS	pxyr1_NCS	4.661	3	0.0186	Yes
pΔXRE_S	pxyr1_S	3.326	3	0.0448	Yes

NCS, no carbon source; S, sophorose

Statistical analysis (2-tailed, 2-sample T-test) of the results from RT-qPCR in Table 1.

Strain_Condition		*t*	df	*P* value	Significant?
Xyr1'(81)_G	TX(WT)_G	60.474	2	0.0003	Yes
Xyr1'(81)_Gly	TX(WT)_Gly	7.322	2	0.0181	Yes
Xyr1'(81)_XN	TX(WT)_XN	33.413	2	0.0009	Yes
Xyr1'(81)_CMC	TX(WT)_CMC	6.120	2	0.0257	Yes

G, glucose Gly, glycerol; XN, xylan; CMC, carboymethyl cellulose

Statistical analysis (2-tailed, 2-sample T-test) of the results from RT-qPCR in Fig. 7A.

Strain_Condition		*t*	df	*P* value	Significant?
QM6a_Δhax_S	QM6a_loxP_S	-4.395	4	0.0117	Yes
QM6a_Δhax_XO	QM6a_loxP_XO	-7.948	4	0.0014	Yes

NCS, no carbon source; S, sophorose; XO, xylose

Statistical analysis (2-tailed, 2-sample T-test) of of the results from RT-qPCR in Fig. 7B.

Strain_Time		*t*	df	*P* value	Significant?
QM6a_Δ*tmus53*_1 h	OEhax1_1 h	1.672	4	0.1699	No
QM6a_Δ*tmus53*_2 h	OEhax1_2 h	-4.380	4	0.0119	Yes

Statistical analysis (2-tailed, 2-sample T-test) of the results from the GoxA reporter gene assay in Fig 7C.

Strain		*t*	df	*P* value	Significant?
pxyr1_OEhax1	pxyr1	3.239	4	0.0317	Yes
pxyr1_OEhax1	pΔXRE	0.292	4	0.7850	No
